# Origin and Evolution of High Nickel Concentrations in Rock Glacier Springs

**DOI:** 10.1021/acsestwater.5c00542

**Published:** 2025-08-26

**Authors:** Simon Seelig, Karl Krainer, Peter Tropper, Michael Pettauer, Albrecht Leis, Thomas Wagner, Giulia Bertolotti, Rudolf Philippitsch, Gerfried Winkler

**Affiliations:** † Department of Earth Sciences, NAWI Graz Geocenter, 27267 University of Graz, Heinrichstrasse 26, Graz 8010, Austria; ‡ Institute of Geology, University of Innsbruck, Innrain 52, Innsbruck 6020, Austria; § Institute of Mineralogy and Petrography, University of Innsbruck, Innrain 52, Innsbruck 6020, Austria; ∥ Institute of Applied Geosciences, NAWI Graz Geocenter, Graz University of Technology, Rechbauerstrasse 12, Graz 8010, Austria; ⊥ JR-AquaConSol GmbH, Steyrergasse 21, Graz 8010, Austria; # Institute for Interdisciplinary Mountain Research; Austrian Academy of Sciences, Innrain 25/3, Innsbruck 6020, Austria; ¶ Austrian Federal Ministry of Agriculture and Forestry, Climate and Environmental Protection, Regions and Water Management, Stubenring 1, Vienna 1010, Austria

**Keywords:** rock glacier, natural acid rock drainage (NARD), solute mobilization, nickel contamination, hydrogeochemical modeling, sulfur isotopes

## Abstract

Global warming has accelerated the degradation of alpine permafrost, significantly altering hydrological and hydrogeochemical processes in mountainous catchments. Among these changes, rock glaciers release spring waters with rising solute concentrations, including nickel levels that significantly exceed drinking water limits. This study investigates the geochemical processes driving solute mobilization in five rock glaciers in the European Alps, focusing on nickel enrichment and transport pathways. Geochemical and petrographic analyses show that high nickel concentrations in spring waters originate from sulfide minerals and their weathering products. Sulfur and oxygen isotope analyses of dissolved sulfate confirm mineral weathering as the primary solute source, while inverse hydrogeochemical modeling accurately reproduces the chemical composition of these spring waters. The hydrogeology of rock glaciers plays a critical role in these processes, as deformation and ice melt expose fresh mineral surfaces, while extended residence times of water in the subsurface create favorable conditions for natural acid rock drainage. Our study demonstrates that rock glaciers are active chemical reactors, enriching alpine streams and lakes with metals and raising concerns about water quality and ecological impacts. This work advances understanding of climate-sensitive metal contamination, informing integrated water management strategies in mountain regions.

## Graphical abstract

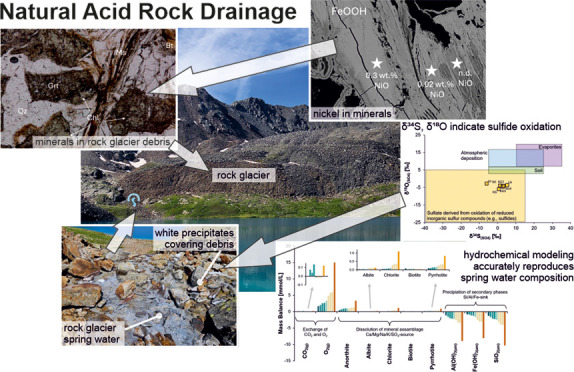

## 1 Introduction

Global warming has significantly increased air temperatures in the European Alps, with a warming rate of ∼0.5 °C per decade between 1991 and 2020, exceeding the global average.^1^ This rapid temperature rise has impacted alpine permafrost, altering hydrology and hydrochemistry in high-altitude catchments.^2–5^ Climate change has caused rising solute concentrations, including dissolved metals, in mountain lakes and streams.^6–10^ This is attributed to either the accelerated weathering of sulfide minerals due to higher ground temperatures and liquid water content, or the release of contaminants stored in melting permafrost ice.^3,5,8,10^ These changes make alpine headwaters particularly vulnerable to climate-induced shifts in water quality.^11–13^

Natural acid rock drainage (NARD) plays a critical role in mobilizing trace metals in alpine environments, especially under warming conditions.^5,14,15^ This process occurs when sulfide minerals, such as pyrite (FeS_2_) and pyrrhotite (Fe_7_S_8_), oxidize in groundwater, leading to acidic water compositions.^16,17^ In rocks with low acid-neutralizing potential the resulting low pH significantly increases the mobility of metals, including nickel, manganese, zinc, and copper, which are commonly found in the crystal structures of sulfide minerals.^17,18^ These metals tend to occur in waters downstream of glaciers or ice-rich permafrost, with their chemical profiles often shaped by the precipitation of secondary Al-hydroxysulfates: aluminum mobilized under low pH conditions forms whitish coatings as the pH approaches neutrality upon mixing with other waters.^5,10,19–23^ Over the past 10–15 years, such whitish crusts characteristic of NARD have been observed in creeks flowing from active rock glaciers in various mountain ranges, highlighting their widespread occurrence.^5,10,21^

Rock glaciers provide crucial freshwater resources in alpine regions that store and release water over different time scales.^11,24–27^ The flow dynamics of rock glacier springs typically exhibit two components: fastflow, driven by rainwater, snowmelt, and ice melt, and baseflow, sustained during winter by the slow release of stored subsurface water.^25–29^ Fastflow dominates during the snowmelt period and summer, peaking after heavy rainfall or intense melt, but declines in autumn as baseflow becomes more prominent.^26,28–30^ Their complex hydrogeological behavior and widespread occurrence make rock glaciers central to understanding water resources in alpine environments.

The hydrochemistry of water emerging from rock glaciers, however, remains poorly understood. Rock glacier springs often exhibit low water temperature (WT), high electrical conductivity (EC), and elevated concentrations of sulfate, magnesium, calcium, and trace metals.^3,7–10,13,18,21,22,29–38^ Over the last decades, alpine lakes fed by rock glacier springs displayed significantly degrading biodiversity and sometimes strong increases in solute concentrations.^6,39–43^ These chemical characteristics are especially pronounced during baseflow-dominated periods in late autumn and winter, raising concerns about their ecological and water quality impacts.^8,29,31,32,43^

Of particular concern is the recurring observation of high nickel concentrations in rock glacier springs.^3,10,13,18,21,22,37,38,43^ These levels frequently exceed the WHO drinking water limit of 70 μg/L.^44^ Nickel is highly toxic and carcinogenic^45^ and has been associated with NARD processes in sulfide-bearing rocks.^21,40,46^ High concentrations of other trace elements, including copper, zinc, manganese, and aluminum, have also been documented in spring waters, permafrost ice, and bedrock.^31,34,35,47,48^ However, the sources of nickel—whether from geochemical weathering, prehistoric atmospheric deposition, or deposition of modern anthropogenic air pollutants—remain a subject of debate.^5–10,13,18,21,32,36,48^ Furthermore, the mechanisms of nickel enrichment and transport are still poorly understood, warranting further investigation.

This study aims to address these knowledge gaps by investigating the origin and evolution of nickel in active rock glacier springs. Using data from five rock glaciers in the European Alps, we explore the geochemical composition of sulfide minerals, assess NARD processes, and evaluate the relationship between rock glacier hydrogeology and nickel mobilization. Our study tests whether high nickel concentrations in rock glacier spring waters originate from rock weathering or from other sources, such as atmospheric deposition. We examine whether a similar chemical process mobilizes metals and dissolves elements across the five study sites, or whether the chemical processes differ from site to site. By developing a quantitative, process-based understanding, we explore implications for water quality and management in mountainous environments.

## 2 Study Sites

The study focuses on five rock glaciers located in the Austrian and Italian Alps (Figure 1). All five rock glaciers contain permafrost ice and some exhibit active movement, with deformation rates reaching up to several millimeters per day.^31,47,49–51^ The rock glaciers are drained by springs with exceptionally high concentrations of trace metals. Geologically, the rock glaciers are derived from bedrocks of the Ötztal-Stubai Complex (ÖSC), a large crystalline complex in the western part of the Austroalpine units. Its metamorphic evolution is summarized in the Supporting Information. This complex is primarily composed of polymetamorphic rocks, with occurrences of sulfide-bearing rocks and localized ore deposits, which may serve as potential sources for metal enrichment.^52,53^ Quartzofelspatic and metapelitic metasediments (mica schists and paragneisses) dominate, with various intercalations of orthogneisses and amphibolites.^54,55^ The following section briefly characterizes each of the five study sites.

**Figure 1:**
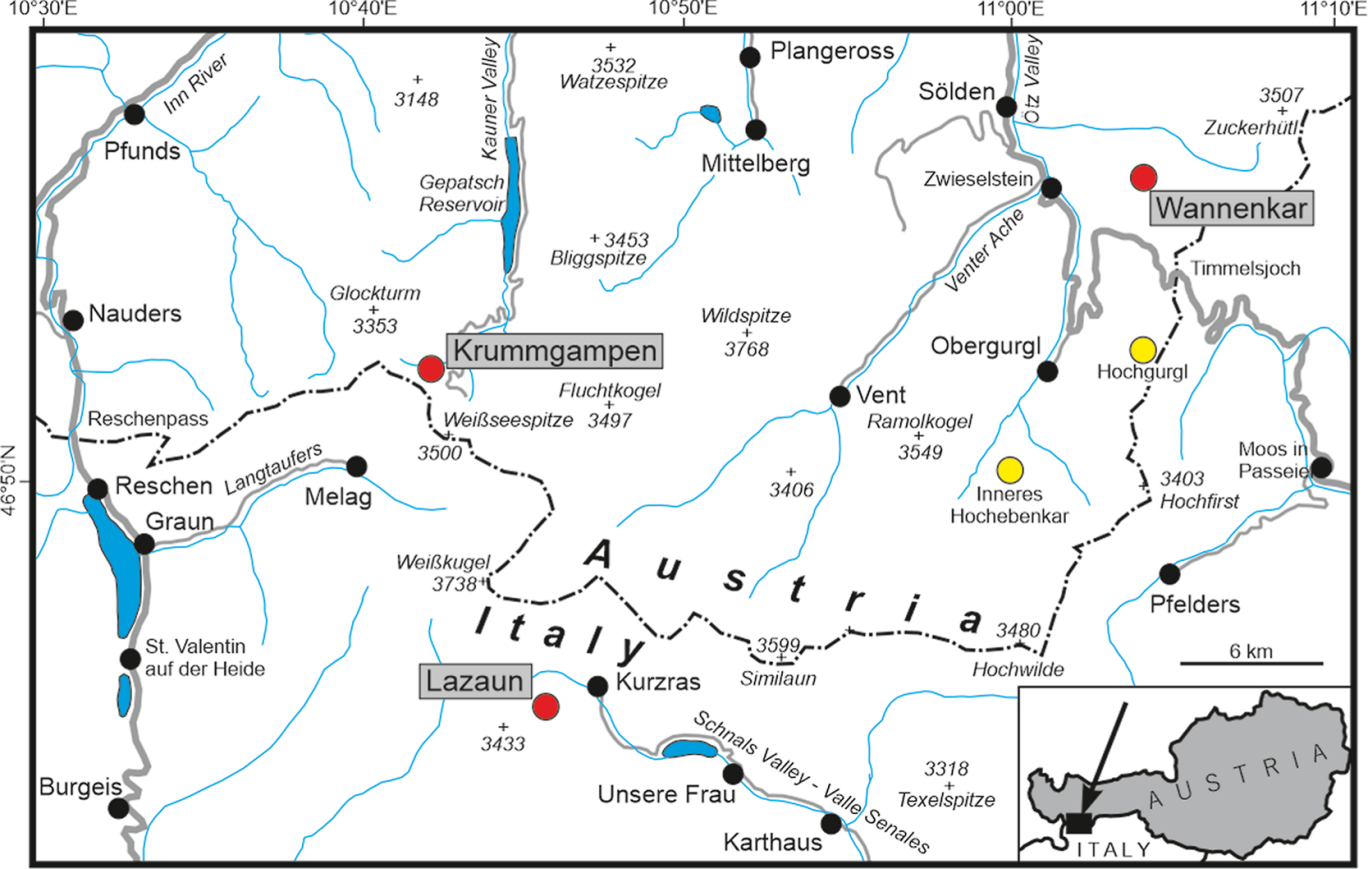
Overview map showing the five study sites. Red dots mark locations where both rock and water samples were collected, while yellow dots indicate sites with water sample collection only.

Lazaun is a NE trending cirque located in the upper Schnals Valley in the southern Ötztal Alps, Italy (Figure 1; 46° 44′ 44″ N, 10° 45′ 14″ E). The bedrock geology consists of an alternating sequence of paragneiss and mica schist, intersected by several fault systems.^31,47^ The cirque hosts an active, tongue-shaped rock glacier that extends 660 m in length, up to 200 m in width, and covers an area of 0.11 km^2^ (Figure 2a,b). It spans elevations from 2480 m a.s.l. at its steep front to 2700 m a.s.l. in the rooting zone, the uppermost part of the rock glacier. The rock glacier has been studied extensively, including two borehole drillings with detailed chemical analyses of the ice cores.^31,47,48,51,56–58^ High nickel concentrations have been repeatedly observed in the rock glacier spring (LA), as well as in several other springs located within the cirque (Figure 2a and Supporting Information Figure S1a).

**Figure 2:**
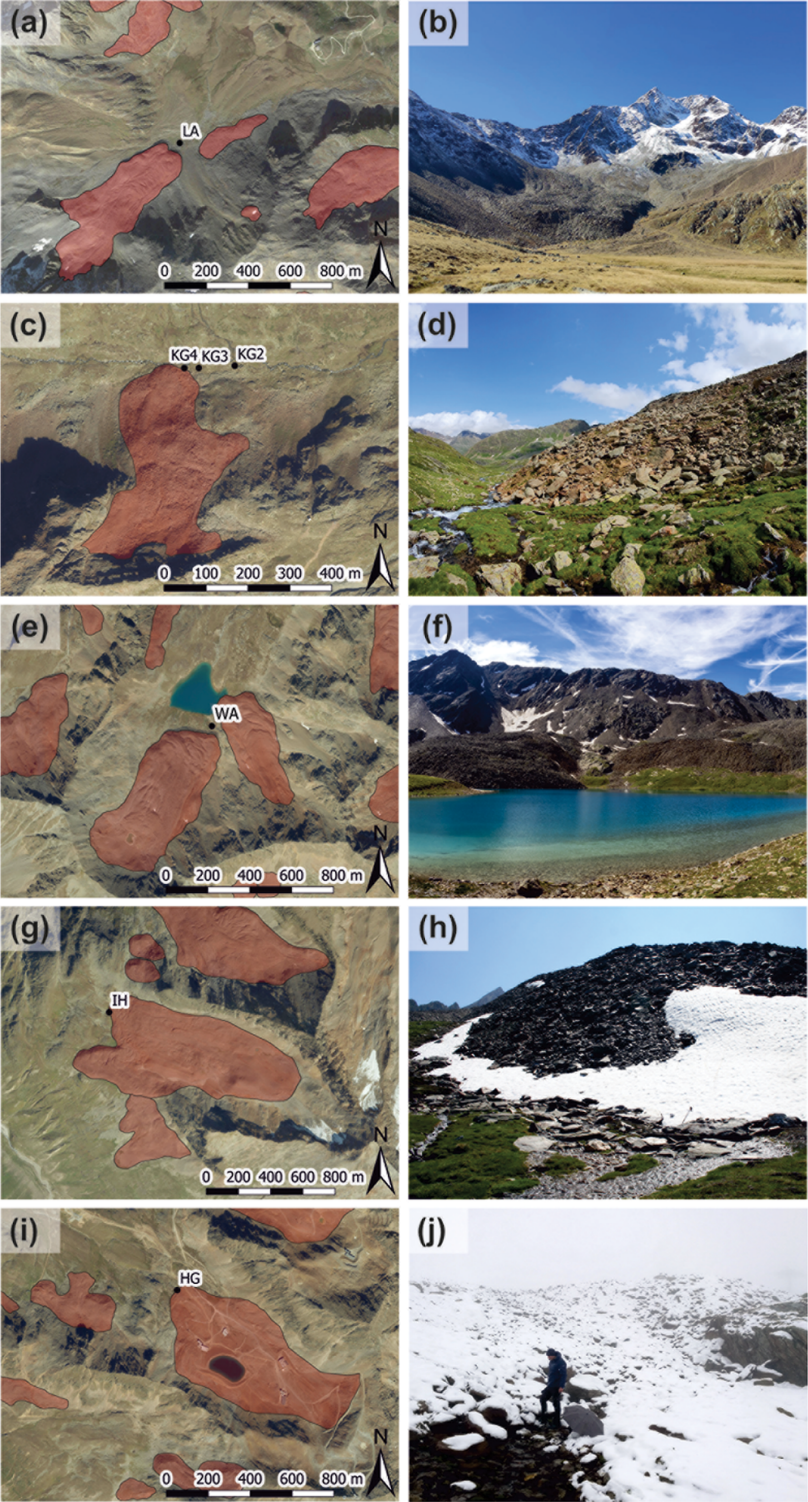
Study sites, (a) Orthophoto of Lazaun (Ötztal Alps, Italy), with rock glaciers outlined in red and the spring sampled for water collection marked as black dot. (b) Photograph of Lazaun and its rock glacier, view is toward SW (Photo: Giulia Bertolotti). (c) Orthophoto of Krummgampen (Ötztal Alps, Austria), with three springs sampled. (d) Photograph of Krummgampen and its rock glacier, view is toward E (Photo: Gerfried Winkler). (e) Orthophoto of Wannenkar (Stubai Alps, Austria), with Lake Wannenkarsee below the two rock glaciers. (f) Photograph of Lake Wannenkarsee and the rock glaciers above, view is toward S (Photo: Gerfried Winkler). (g) Orthophoto of Inneres Hochebenkar (Ötztal Alps, Austria). (h) Photograph of the rock glacier front at Inneres Hochebenkar, view is toward E (Photo: Karl Krainer). (i) Orthophoto of Hochgurgl (Ötztal Alps, Austria). (j) Photograph of the rock glacier and its sampled spring at Hochgurgl, view is toward S (Photo: Gerfried Winkler). Rock glacier outlines are based on Wagner et al.^49^ and Zischg et al.,^60^ orthophotos are provided by Open Government Data Austria (www.data.gv.at).

Krummgampen is a 3 km-long, east–west-trending side valley of the upper Kauner Valley in the Ötztal Alps, Austria, situated between 2400 and 3300 m a.s.l. elevation (Figure 1; 46° 52′ 23″ N, 10° 42′ 2″ E). The bedrock is composed of paragneiss, orthogneiss, amphibolite, and mica schist of the Ötztal Unit, crosscut by numerous faults, along which the rocks are heavily fractured.^31,55^ A north-facing rock glacier deflects the stream at 2465 m a.s.l (Figure 2c,d and Supporting Information Figure S1b). Three springs emerge east of the front (KG2–4). Low summer WT (<1.5 °C) suggest permafrost influence, supported by geophysical and hydrological data.^4^ Spring water shows high EC and elevated nickel concentrations.^31,32,38^ Whitish crusts form on the boulders of the creek, immediately downstream from its confluence with the waters of the rock glacier springs (Supporting Information Figures S2 and S3). Detailed chemical and mineralogical analyses of these mineral coatings are provided by Thies et al.^22^

Wannenkar cirque, located in Windachtal in the Stubai Alps, Austria, hosts a mountain lake (Wannenkarsee, 2639 m a.s.l.) fed by two prominent rock glaciers (Figure 1; 46° 56′ 34″ N, 11° 4′ 27″ E). One of them terminates ∼80 m south of the lake with several springs at its front, while the other extends directly into the eastern part of the lake (Figure 2e,f and Supporting Information Figure S1c). The bedrock geology consists of paragneiss and mica schist of the Ötztal Unit.^31,59^ Several springs draining the rock glaciers are characterized by high EC, low pH, and high concentrations of aluminum, manganese, nickel, and cobalt, especially under baseflow conditions.^31,59^ Of these, the spring labeled WA in Figure 2e is analyzed in this study. Whitish crusts form on the boulders downstream from the rock glacier springs as well as downstream from the lake (Supporting Information Figures S4 and S5).

Inneres Hochebenkar is an alpine cirque in the Hochebenkamm, Ötztal Alps, Austria (Figure 1; 46° 49′ 34″ N, 11° 0′ 34″ E). An active rock glacier covers its lower sections (Figure 2g,h). This two-lobed rock glacier spans 0.57 km^2^, measuring 1268 m in length and 597 m in width, with its front at 2648 m a.s.l. and its rooting zone at 2948 m a.s.l. (Supporting Information Figure S1d).^50^ Numerous springs emerge at its steep front, exhibiting elevated concentrations of aluminum, iron, manganese, and nickel. Among these, the spring labeled IH in Figure 2g is the focus of this study. The bedrock geology features mica schists and paragneisses of the Ötztal Unit, with localized fault zones resulting in heavily crushed rock.^50^

The study site Hochgurgl is situated in the Ötztal Alps, Austria, within a cirque located above the Gurgler Valley (Figure 1; 46° 53′ 20″ N, 11° 4′ 20″ E). This cirque hosts a rock glacier extending 994 m in length and up to 489 m in width, covering an area of 0.33 km^2^ (Figure 2i,j). The rock glacier spans elevations from 2646 to 2905 m a.s.l. and features a spring at its front (HG). The cirque also includes an artificial lake, used for snow production in winter, located on top of the rock glacier. The underlying bedrock comprises metagranite gneisses of the Ötztal Unit.

## 3 Methods

### 3.1 Geochemical and Petrographic Analyses

To understand the relationship between lithological composition and spring water chemistry, representative bedrock samples were collected near the rock glacier fronts at Lazaun, Krummgampen, and Wannenkar. Their bulk chemical composition was determined by XRF for Lazaun and Krummgampen, while thin sections for petrographic and electron microprobe analysis were prepared for all three sites. Wavelength-dispersive (WDS) and energy-dispersive (EDS) microanalysis was performed using JEOL JXA-1 SUPERPROBE and JEOL JXA-iSP100 instruments at the Institute of Mineralogy and Petrography, University of Innsbruck, under measurement conditions of 15 kV accelerating voltage and 10 nA beam current, with a focused beam diameter of 1 μm. Peak and background counting times were 20 and 10 s, respectively. Natural and synthetic standards used for calibration included quartz (Si), corundum (Al), orthoclase (K), diopside (Ca), Rutile (Ti), Ni-olivine (Ni), rhodonite (Mn), Almandine (Fe), jadeite, periclase (Mg) and troilite (S). Standardless EDS analysis used a counting time of 60 s, with quantification based on the fundamental-parameter approach and normalization to 100 wt %.

### 3.2 Hydrogeochemical and Isotopic Analyses

#### 3.2.1 Sampling and Laboratory Analyses

To investigate the hydrochemical characteristics of the rock glacier springs, sampling campaigns were conducted in late June and between August 30 and September 1, 2021. Major and trace elements, as well as isotopic ratios of sulfur and oxygen in sulfate, were analyzed. For all sampling events, water was collected in 100 mL and 2 × 1 L PET bottles. Additionally, the Lazaun rock glacier spring was sampled multiple times in 2007 to examine seasonal variations in water composition, with samples collected in January, August, and October. Field parameters, including EC, WT, pH, and oxygen content, were measured using a multiparameter hand-held device (WTW Multi 3430). Laboratory analyses were performed at the Institute of Applied Geosciences, Graz University of Technology, using inductively coupled plasma optical emission spectrometry for measuring trace elements (ICP-OES; PerkinElmer OPTIMA 8300 DV with an autosampler AS 930 with an analytical uncertainty ±3% and a limit of detection (LOD) of 1 μg/L for Co, Cu, Fe, Li, Mn and Zn, of 5 μg/L for Al and Ni, and of 30 μg/L for Si). Major chemical compounds (Ca^2+^, Cl^–^, K^+^, Mg^2+^, Na^+^ and SO_4_^2–^) were measured with ion-exchange chromatography (Dionex IC S 3000, IonPac, AS19 and CS16 column; analytical uncertainty ±3%; LOD 0.1 mg/L). The carbonate-alkalinity (HCO_3_^–^) was analyzed by a potentiometric titration using a Schott TitroLine alpha-plus titrator with 0.02 M HCl (analytical uncertainty ±2%). For the Lazaun spring samples, only major elements were analyzed. Given the well-documented high nickel concentrations at this spring (up to 66 μg/L),^31,47^ these samples were included in the analysis to provide further insights.

Spring water sulfate was precipitated as BaSO_4_ to analyze its sulfur and oxygen isotope composition. δ^34^S_(SO4)_ values were measured by elemental analyzer isotope ratio mass spectrometry (EA-IRMS) and reported relative to the Vienna Cañon Diablo Troilite (VCDT) standard.^61^ δ^18^O_(SO4)_ values were determined by high-temperature conversion isotope ratio mass spectrometry (HTC-IRMS) and reported relative to the Vienna Standard Mean Ocean Water (VSMOW).^62^

#### 3.2.2 Hydrogeochemical Modeling

The PHREEQC V3 software, utilizing the phreeqc.dat and carbfix.dat databases, was employed to calculate ion balance errors, aqueous speciation, and saturation indices (SI) for key primary and secondary minerals.^63,64^ These included carbonate (calcite, dolomite), silicate (anorthite, albite, K-feldspar, quartz, muscovite), sulfate (gypsum), and sulfide minerals (pyrite, pyrrhotite), along with secondary precipitates such as kaolinite, allophanes (short-range ordered aluminosilicate phases),^65,66^ basaluminite,^23,67,68^ and amorphous phases of SiO_2_, Al­(OH)_3_, and Fe­(OH)_3_. The internal partial pressure of CO_2_ was also calculated. Charge balance errors for the samples taken at Krummgampen, Wannenkar, Inneres Hochebenkar, and Hochgurgl were within ±1%, whereas the samples taken at Lazaun exhibited higher errors ranging from −6.2% to +3.8%.

Inverse modeling was used to trace the evolution of rain- or snowmelt-derived water to the observed chemistry of rock glacier springs. Given the inherent data limitations, a model focusing on major ion chemistry and dominant rock-forming minerals provided a first-order approximation of the key geochemical processes, as metal mobilization is closely linked to weathering and oxidative dissolution processes within the NARD framework.^5,17,69,70^ Primary minerals were defined as dissolving only, while secondary phases, represented by amorphous SiO_2_, Al­(OH)_3_, and Fe­(OH)_3_, were allowed to precipitate only. CO_2_ and O_2_ exchange with the atmosphere were unrestricted, with partial pressures fixed at atmospheric levels. The mineral assemblage and stoichiometry of selected minerals was derived from petrographic analysis of the Krummgampen rock glacier host rock, which closely resembles that of the other sites, and included anorthite (CaAl_2_Si_2_O_8_), albite (Na_2_AlSi_3_O_8_), biotite (KMg_1.2_Fe_1.4_Al_1.8_Si_2.6_O_10_(OH)_2_), chlorite (Mg_3_Fe_2_Al_2_Si_3_O_10_(OH)_8_), and pyrrhotite (Fe_7_S_8_). Mineral dissolution reactions and stoichiometry were parametrized using the carbfix.dat database, while the compositions for biotite, chlorite, and pyrrhotite were inferred from microprobe measurements. The full modeling details, including the model code, are provided in the Supporting Information.

## 4 Results

### 4.1 Geochemical and Petrographic Analyses

#### 4.1.1 Mineral Assemblages

The metamorphic rocks at Lazaun, Krummgampen, and Wannenkar mainly consist of garnet-bearing mica schists and paragneisses with intercalated amphibolites. Their chemical composition reflects typical low-Ca pelitic characteristics with SiO_2_ ranging from 45–64 wt %, Al_2_O_3_ 18–23 wt %, Fe_2_O_3_ 7–14 wt %, MnO 0.07–0.1 wt %, CaO 0.5–1.4 wt %, Na_2_O 1.7–2.8 wt %, K_2_O 2.8–4.6 wt %, and TiO_2_ 0.8–1.7 wt %.

The mineral assemblage in the mica schists is garnet + staurolite + biotite + muscovite + plagioclase + quartz ± kyanite ± chloritoid (Figure 3a). Monazite, xenotime, allanite, tourmaline, zircon, and sulfide minerals (chalcopyrite, pyrrhotite, pyrite) occur as accessory phases, where monazite and xenotime show corrugated grain boundaries in the Krummgampen samples. At Wannenkar and Lazaun, staurolite shows replacement by chloritoid along fractures and grain boundaries as well as complete pseudomorphs after chloritoid and white micas (Figure 3a). At Krummgampen, staurolite is replaced only by chlorite. Garnet is frequently replaced by chlorite in the samples from Lazaun (Figure 3b). Sulfide minerals such as pyrrhotite were found to be chemically altered and partially replaced by secondary Fe-hydroxides, likely through oxidative weathering^71^ (Figure 3c–f). The amphibolites contain the mineral assemblage hornblende + biotite + epidote + plagioclase + quartz. As accessory phases, rutile, ilmenite, titanite, and sulfide minerals (pyrite, pyrrhotite) occur. Again, sulfide minerals are almost completely replaced by Fe-hydroxides (Figure 3).

**Figure 3:**
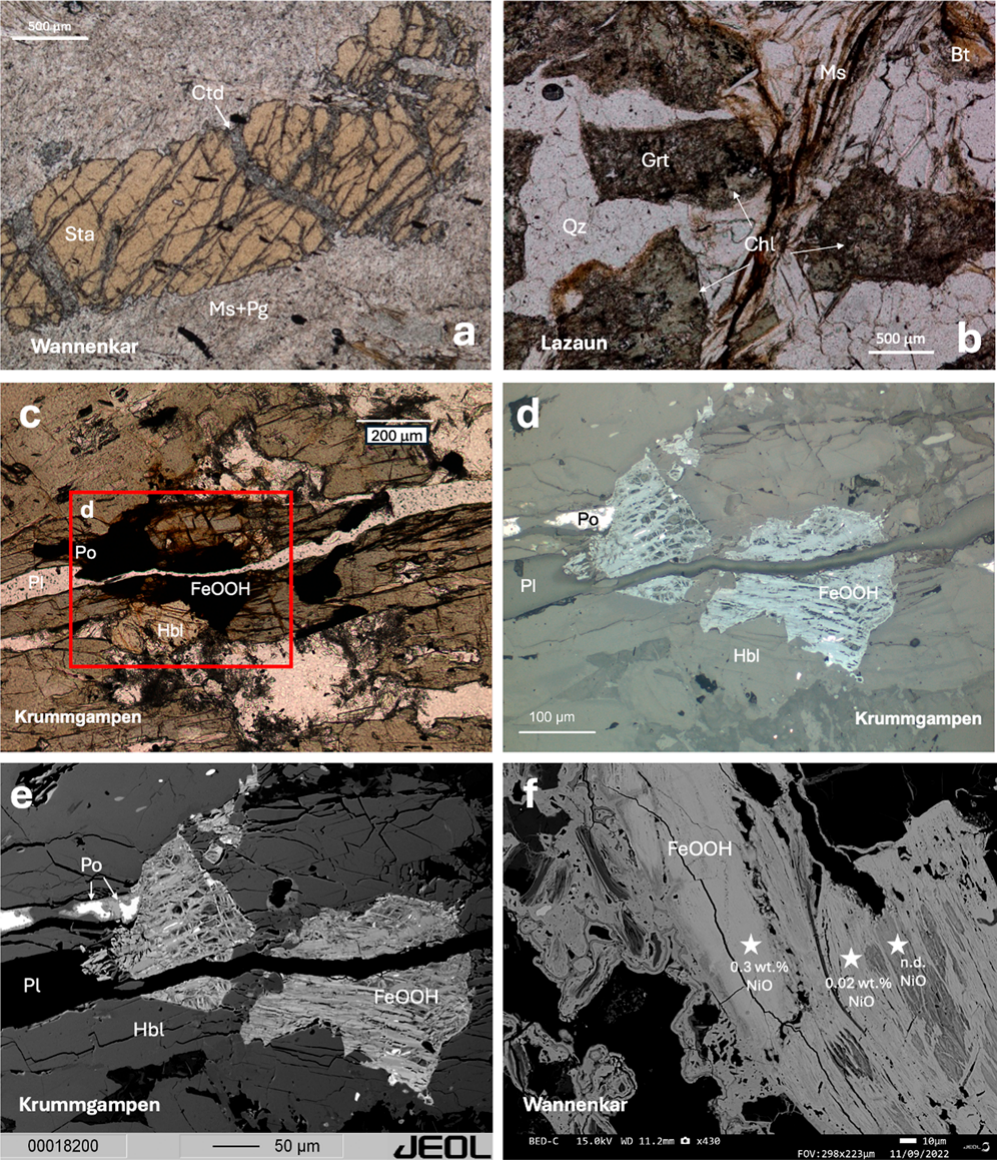
Polarized photomicrographs (a–c) of a mica schist from Wannenkar (a), a paragneiss from Lazaun (b) and an amphibolite from Krummgampen (c). The sample from Wannenkar in (a) shows partial replacement of staurolite (Sta) by chloritoid (Ctd) along fractures in staurolite. The fine-grained matrix surrounding staurolite consists of white micas such as muscovite and paragonite. The Lazaun sample in (b) shows alteration of garnet (Grt) by chlorite (Chl) in a matrix of quartz (Qz), muscovite (Ms), and biotite (Bt). The Krummgampen amphibolite (c) shows the silicate mineral assemblage of hornblende (Hbl) and partly altered plagioclase (Pl). The sulfides and Fe-hydroxides appear dark brown and black. Figure (d) represents the reflected light close-up microscope image of the red rectangle in Figure (c), which allows a clear discrimination between the sulfide mineral pyrrhotite (Po) and the Fe-hydroxide replacement minerals (FeOOH). Panels (e,f) show Backscattered Electron (BSE) images of the amphibolite sample from Krummgampen (e) and pyrrhotite (Po) pseudomorphs of Fe-hydroxides (FeOOH) in a paragneiss from the Wannenkar (f). Figure 3 (e) represents the BSE image of the red close-up rectangle of Figure (c). Stars in (f) mark analysis positions and corresponding NiO contents in the FeOOH pseudomorph; n.d. = not detected, and values <0.1% are near the EDS detection limit and should be interpreted qualitatively.

#### 4.1.2 Nickel in Minerals

The element contents of sulfides, oxides, and silicates, determined through EDS and WDS, provide insights into their concentrations across different mineral groups (Supporting Information Table S1 and Figure S6). Pyrrhotites exhibit the highest nickel concentrations, ranging from 0.1 wt % to 1.35 wt % Ni, with peak values observed at Wannenkar. Chalcopyrite is common in samples from Wannenkar and Lazaun, occasionally containing up to 0.1 wt % Ni. Oxidative weathering of sulfide minerals leads to the formation of Fe-hydroxides (e.g., goethite), which contain up to 1 wt % NiO (Figure 3c–f), as observed at Krummgampen, where Fe-hydroxide formation occurs in three stages with decreasing nickel contents. The silicate minerals generally show very low NiO contents of ≤0.05 wt %. The highest were found in hornblendes and chlorites. Garnet contains up to 0.03 wt %, muscovite and biotite also contain small amounts (<0.01 wt %). In summary, nickel is most enriched in pyrrhotites, while silicates contain only minor amounts.

### 4.2 Hydrogeochemical and Isotopic Analyses

#### 4.2.1 Spring Water Composition

The physical and chemical properties of spring water samples display specific characteristics. WT ranged from 0.6 to 2.5 °C, pH from 4.98 to 7.17, and EC from 171 to 1188 μS/cm. However, subsurface conditions may differ, with WT potentially lower where water is in contact with ice, EC higher in concentrated porewaters, and pH significantly lower in zones of sulfide oxidation. Dissolved oxygen levels were near atmospheric saturation (92.7 to 98.0%). Na^+^, K^+^, and Cl^–^ concentrations were generally low, at ≤3.2 mg/L, ≤3.1 mg/L, and ≤1.2 mg/L, respectively. Mg^2+^ and Ca^2+^ showed considerable variation, with Mg^2+^ ranging from 4.8 to 81.1 mg/L, and Ca^2+^ from 18.4 to 132.3 mg/L. SO_4_^2–^ concentrations ranged from 70.1 to 637.0 mg/L, while HCO_3_^–^ concentrations spanned 0.6 to 9.2 mg/L. Dissolved Si varied between 1.0 and 6.6 mg/L. Al concentrations ranged from <1 to 2592 μg/L, while total Fe concentrations were low (≤2 μg/L). Of particular concern was Ni, with concentrations between 105 and 295 μg/L (except for one value of 38 μg/L), far exceeding the Austrian drinking water limit of 20 μg/L and the limit of 70 μg/L defined by WHO.^44,72^ Mn, Cu, and Zn also showed elevated concentrations, reaching up to 445 μg/L, 32 μg/L, and 162 μg/L, respectively. Co concentrations reached a maximum of 40 μg/L, Li concentrations peaked at 18 μg/L. Complete results are provided in Supporting Information Table S2.

To determine whether these values are typical for rock glacier springs or site-specific, we compared them with data from 195 rock glacier springs, serving as a baseline reference for spring water chemistry.^13^ The one-sided rank sum test^73,74^ revealed significantly elevated concentrations of SO_4_^2–^, Al, Cu, Mn, Ni, and Zn (*p* < 0.01), substantially exceeding baseline values (Supporting Information Table S3 and Figure S7). Metal concentrations were particularly high, with Ni showing an average increase of +226 μg/L (*p* = 2.3 × 10^–5^), Al + 214 μg/L (*p* = 5.9 × 10^–4^), Zn + 139 μg/L (*p* = 4.2 × 10^–5^), Mn + 66 μg/L (*p* = 1.1 × 10^–5^), and Cu + 17 μg/L (*p* = 3.6 × 10^–5^). As a byproduct of sulfide mineral oxidation, SO_4_^2–^ exhibited an increase of +117 mg/L (*p* = 2.8 × 10^–5^). K^+^, Mg^2+^, and Ca^2+^ were slightly elevated, Na^+^, Si, and total Fe remained close to the baseline reference, and Cl^–^ and HCO_3_^–^ showed modest reductions (Supporting Information Table S3). Geographically, elevated concentrations were limited to specific springs, with contaminated and inconspicuous sites interspersed, pointing to site-specific controls on water chemistry.

The sulfur and oxygen isotope compositions of sulfate in spring water were analyzed to investigate its sources. Measured δ^34^S_(SO4)_ values ranged from −6.0‰ to +5.2‰ (mean +1.3‰), while δ^18^O_(SO4)_ values ranged from −5.9‰ to −2.8‰ (mean −4.0‰), summarized in Supporting Information Table S4. Figure 4 compares these isotopic signatures with typical values from various sulfate sources, such as atmospheric deposition and the oxidation of inorganic sulfur compounds during mineral weathering (see Supporting Information). As indicated by low δ^34^S_(SO4)_ and δ^18^O_(SO4)_ values (yellow box in Figure 4), sulfate in the seven rock glacier springs mainly derives from sulfide mineral oxidation.

**Figure 4:**
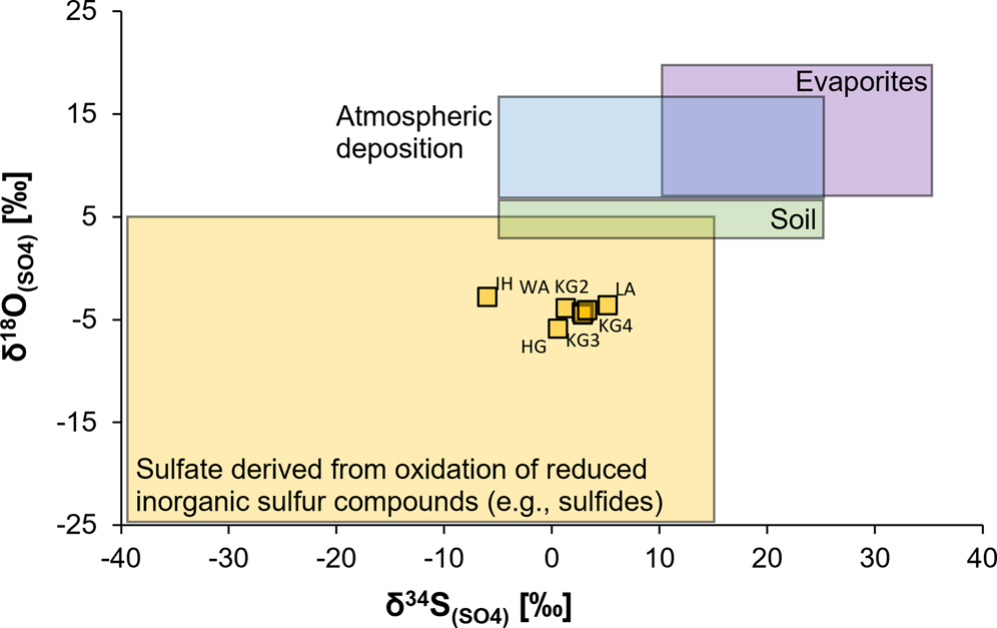
Oxygen and sulfur isotopic composition of sulfate in sampled rock glacier spring waters (gold squares; the error bars are within the symbol size). δ^34^S_(SO4)_ values are plotted along the abscissa relative to VCDT, while δ^18^O_(SO4)_ values are shown on the ordinate with respect to VSMOW. For comparison, typical ranges in δ^34^S_(SO4)_ and δ^18^O_(SO4)_ for various sources are indicated (adapted from Krouse and Mayer,^83^ see Supporting Information). HG = Hochgurgl, IH = Inneres Hochebenkar, KG2–4 = Krummgampen, LA = Lazaun, WA = Wannenkar.

For four of the seven rock glacier springs, sufficient spring water samples were available^38,75,76^ to differentiate between atmospheric and water-derived oxygen sources in sulfate (KG2, KG3, KG4, LA). Due to the large δ^18^O contrast between atmospheric O_2_ (+23.88‰)^77^ and H_2_O (varying from −14.11‰ to −13.34‰ between the springs, Supporting Information Table S4), and minimal isotope exchange between dissolved sulfate and water even at low pH, the δ^18^O_(SO4)_ signature can be used to evaluate the relative participation of two reaction pathways where either O_2_ or Fe^3+^ acts as the oxidant (see Supporting Information).^78–81^ The results indicate that 68–75% of the oxygen in sulfate derives from Fe^3+^-mediated oxidation, indicating that ferric iron is the dominant oxidant in sulfide weathering within these rock glaciers (Supporting Information Table S4). While this estimate considers atmospheric O_2_ as an alternative oxidant, it does not capture potential contributions from other sources such as reactive oxygen species (e.g., H_2_O_2_), which may also play a role in sulfide oxidation.^82^

#### 4.2.2 Hydrogeochemical Modeling

Thermodynamic calculations reveal consistent trends in mineral stability, dissolution, and secondary mineral formation across the analyzed rock glacier springs. Supporting Information Table S5 summarizes the saturation indices (SI) for rock-forming minerals and possible secondary phases. Most spring waters were undersaturated with respect to calcite, dolomite, gypsum, and feldspar minerals. Quartz was near equilibrium (SI = 0.07 ± 0.31), whereas muscovite showed clear supersaturation, preventing further dissolution. Pyrite and pyrrhotite were strongly undersaturated, suggesting their potential for dissolution under oxidizing conditions. Kaolinite, a typical secondary clay mineral, was supersaturated in all cases. The amorphous Si-phase was undersaturated, with SI-values slightly lower than those of quartz. Al- and Fe-phases varied from undersaturation to supersaturation across samples. Both allophane and basaluminite were generally supersaturated, but showed undersaturation in some samples. Overall, the SI values suggest a corrosive environment for carbonate and silicate rocks, promoting feldspar dissolution and the formation of secondary minerals, including clay minerals through (semi)­amorphous precursors like allophane and hisingerite, as well as the precipitation of basaluminite.^65^

The mass balance results are depicted in Figure 5, with the underlying hydrogeochemical model developed using the host rock composition of the Krummgampen rock glacier. To emphasize the core processes of NARD, the inverse model was simplified to key minerals accounting for major dissolved ions, reducing model ambiguity while capturing essential water–rock interactions and ensuring consistent application across all springs with reasonable uncertainty (see Supporting Information). The uncertainty was ≤1% for most samples, except at Lazaun, where uncertainties were slightly higher (3.8%, 6.2%, and 5.4% for samples LA_Jan_, LA_Aug_, and LA_Oct_, respectively). The results revealed minimal CO_2_ exchange (±6 mg/L) and a substantial oxygen demand (up to 479 mg/L). Anorthite emerged as the dominant contributor to dissolved ions, with contributions of up to 917 mg/L, followed by chlorite with up to 505 mg/L. Other silicate minerals, like albite and biotite, contributed less than 37 mg/L. The demand for oxygen (up to 479 mg/L) was tied to the oxidation of pyrrhotite (up to 540 mg/L) considering the molar ratio of the dissolved SO_4_^2–^ species (S: 4O = 1.12 with *R* ^2^ = 1.00).

**Figure 5:**
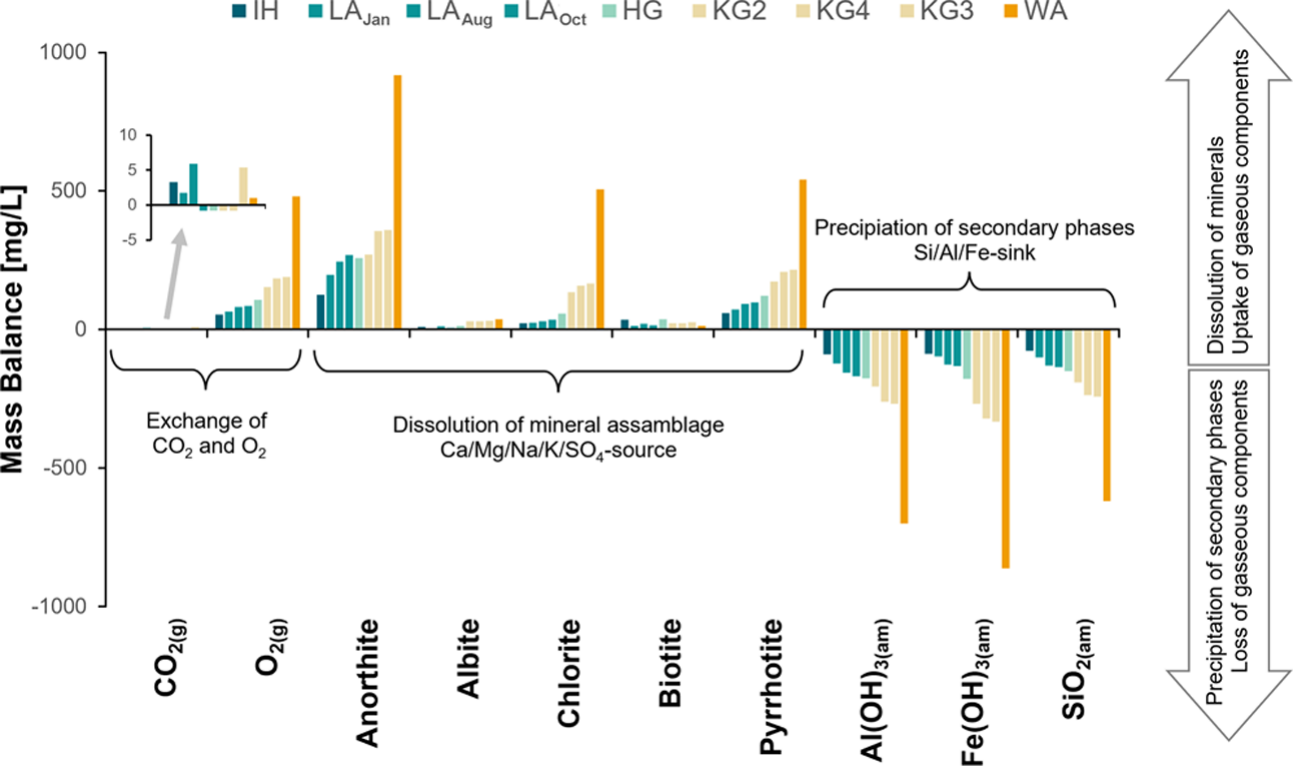
Results of the mass balance of the inverse model calculated for all seven rock glacier spring samples, with the same rock assemblage derived from the Krummgampen site. Positive mass balance values indicate dissolution or gas uptake, negative values indicate precipitation or degassing. The samples are arranged in order of increasing oxygen consumption from left to right. Note that the oxygen demand represents both oxidation mechanisms of pyrrhotite/pyrite (i.e., oxidation by dissolved O_2_ and Fe^3+^). The formation of secondary phases is summed up by the amorphous phases of Si, Al and Fe forming various phases such as allophane, Fe-hydroxides, and hisingerite. IH = Inneres Hochebenkar, LA = Lazaun (sampled in January, August, and October), HG = Hochgurgl, KG2–4 = Krummgampen, WA = Wannenkar.

## 5 Discussion

In mountainous catchments, elevated dissolved metal concentrations are not uncommon in springs, creeks, and lakes, particularly in cryospheric environments like rock glacier springs, where solute levels can be among the highest recorded.^5,21^ While previous studies identified NARD as the primary mechanism behind these solute enrichments through correlation analyses and laboratory experiments, this study provides the first quantitative assessment of the entire pathway from identified source minerals over their oxidative dissolution to spring water hydrochemistry. Dual-isotope analysis of δ^34^S_(SO4)_ and δ^18^O_(SO4)_ identified weathering of rocks containing sulfide minerals as the dominant source of sulfate in spring waters and constrained the oxidation mechanism. Hydrogeochemical modeling effectively reconstructed the spring water composition, quantified mineral saturation states and mass balances, and revealed key processes governing metal mobilization across the studied rock glaciers.

Figure 6 illustrates the conceptual model of NARD in rock glaciers, explaining the observed high sulfate concentrations, low pH, and elevated metal levels at the springs. While the measured pH values at the spring outlets range from 4.98 to 7.17, it is likely that sulfide oxidation and Fe^3+^ mobilization occur under more acidic conditions in the subsurface, where pH may be significantly lower before dilution along the flow path. The key sources of dissolved ions are host rock minerals, like albite and anorthite supplying Na^+^, Ca^2+^, Al, and Si, while chlorite and biotite provide K^+^, Mg^2+^, Fe, Al, and Si. Oxidation of sulfide minerals such as pyrrhotite and pyrite by oxygen (O_2_) and ferric iron (Fe^3+^) produces sulfuric acid, lowering pH and increasing sulfate concentrations. The aqueous Na^+^, K^+^, Ca^2+^, and Mg^2+^ ions behave conservatively at these low pH conditions, remaining largely dissolved. The low concentrations of Si, Al, and Fe require effective sinks for these elements. Slight pH increases readily remove Fe from the solution due to the oxidizing conditions, either precipitating as amorphous to poorly crystalline Fe-(hydr-)­oxides or forming compounds such as hisingerite.^65^ Si is fixed by adsorption and coprecipitation within these precipitates. Moreover, slight pH increases can induce the formation of short-range-ordered allophane phases, which act as a sink for both Al and Si.^65^ The concentration of Si is therefore mainly controlled by the coprecipitation of Al/Si- and Fe/Si-phases, which act as precursors for secondary clay minerals like kaolin- or smectite-group sheet-silicates. These precipitates, potentially including phases such as basaluminite, form the whitish mineral coatings observed on creek boulders in some of the study sites (Supporting Information Figures S2–S5), in line with chemical and structural analyses.^21–23^ Although not explicitly accounted for in the hydrogeochemical model, the prevailing conditions also promote the mobilization of trace metals, including Ni. As observed, Ni is primarily hosted in sulfide minerals, with minor contributions from other rock-forming phases, and is therefore released as a byproduct of the NARD process.

**Figure 6:**
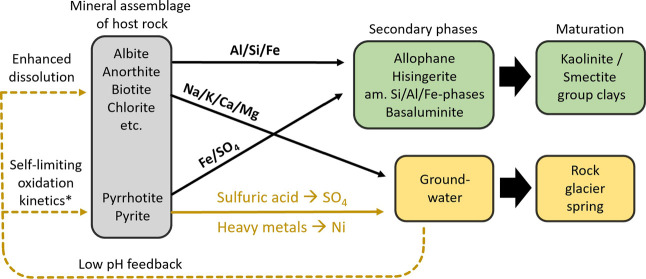
Conceptual model illustrating the evolution of the chemical composition of rock glacier springs through natural acid rock drainage (NARD). The host rock mineral assemblage is presented in terms of the most relevant minerals, while secondary minerals and their precursors are also indicated. The model highlights dominant solute pathways and pH feedbacks. *The term “self-limiting oxidation kinetics” refers to the general pH-dependence of sulfide oxidation rates;^84^ however, additional mechanisms such as oxidation by atmospheric O_2_ or reactive oxygen species (ROS) may also operate under near-neutral conditions.

The acidic conditions resulting from sulfide oxidation trigger three feedback mechanisms that influence hydrogeochemical processes. First, the low pH is enhancing the dissolution of silicate host rock minerals, increasing the release of dissolved ions such as Ca^2+^, Mg^2+^, Na^+^, K^+^, and Si into the water.^85^ This process not only contributes to the overall solute load but also buffers the pH, partially neutralizing the acidity. However, the extent of this buffering effect depends on reaction kinetics as well as on the mineralogy of the host rock and the residence time of water in contact with reactive surfaces. Second, under acidic conditions below pH 4, as likely present in microsites of active sulfide weathering, Fe^3+^-mediated sulfide oxidation can become the dominant reaction pathway.^78,79,86,87^ This process often proceeds quasi-autocatalytically due to the enhanced solubility and reactivity of Fe^3+^ under such low-pH conditions. Although the reaction may become self-limiting due to restricted Fe^3+^ regeneration or the formation of passivating layers such as elemental sulfur,^81^ the observed oxygen isotope signature in sulfate indicates that Fe^3+^-mediated pathways likely account for over two-thirds of total oxidation. While our data suggest that Fe^3+^-mediated pyrite oxidation may contribute significantly to SO_4_^2–^ formation, alternative pathways, including direct oxidation by atmospheric O_2_ and incorporation of oxygen from reactive oxygen species (ROS), cannot be excluded, particularly under near-neutral pH conditions.^82^ Third, sulfide oxidation may be limited by the high solubility of Fe^3+^ at pH < 3, conditions not observed in spring waters but likely present in the microenvironment around oxidizing Fe-sulfide grains.^79,81,88^

The hydrogeochemical model demonstrates that NARD occurs consistently across all seven springs and five rock glacier sites and dominates the water chemistry. Despite its simple design, the model effectively captures the observed composition of spring waters with reasonable accuracy. The low pH resulting from this process enhances the mobility of metals typically incorporated as trace elements within the crystal structures of sulfides like pyrrhotite. The hydrogeology of rock glaciers facilitates these processes, turning them into chemical reactors that enrich alpine lakes and creeks with metals. Three critical factors underpin this role, establishing a causal connection between the observed hydrochemistry and rock glacier hydrogeology.

First, rock glaciers provide oxygen to initiate sulfide oxidation through their well-ventilated internal structure. The inverse model implies that the oxygen demand is between 52 and 479 mg/L, although it does not differentiate between O_2_-driven and Fe^3+^-mediated reaction mechanisms. Nevertheless, especially in the initial stages, a sufficient supply of dissolved O_2_ is necessary to start the oxidation process. The heterogeneous ice-debris mixture forming the frozen rock glacier cores creates extensive pathways for air ventilation, as evidenced by borehole drillings,^47,89,90^ modeling studies,^91,92^ and geophysical investigations.^93,94^ These findings support and extend the conceptual model of rock glacier NARD proposed by Moradi et al.,^10^ which highlights the critical role of oxygen availability for sulfide oxidation in rock glaciers, by additionally emphasizing the importance of Fe^3+^ as a key oxidant under acidic conditions and relating both processes to rock glacier hydrogeology.

Second, continuous deformation, grain crushing, and permafrost ice melt within rock glaciers expose fresh, chemically active mineral surfaces and increase the specific surface area available for reactions. Borehole results at Lazaun show distinct zones with significantly enriched metal concentrations and high EC values.^48^

Third, prolonged subsurface water residence times on the order of months^11^ facilitate prolonged interaction with reactive mineral phases. Longer residence times allow for sustained contact between water and mineral surfaces, promoting the enrichment of solutes in water. The heterogeneous structure of rock glaciers, with fine-grained zones storing water and coarse-grained areas enabling oxygen supply, facilitates sulfide oxidation, which requires long-term storage to allow the gradual buildup of reactive components and sustaining oxidation processes over extended periods.^18,21^

In summary, the interaction between water, oxygen, and freshly exposed mineral surfaces intensifies solute mobilization in active rock glaciers, while their water storage capacity and prolonged subsurface water residence times enable extensive chemical reactions. Together, these factors highlight the critical role of rock glacier hydrogeology in driving the release of solutes, including dissolved metals, into downstream environments.

## 6 Conclusions

The results of this study enhance our understanding of the processes driving natural acid rock drainage (NARD) in rock glaciers and its impact on spring waters. By combining geochemical and petrographic analyses, isotope analyses, and hydrogeochemical modeling, we were able to quantitatively reconstruct the oxidative dissolution and mobilization processes that result in elevated concentrations of dissolved metals such as nickel in alpine spring waters. This approach allowed us to trace the path of these solutes from their source in sulfide minerals to their release from rock glaciers through spring waters. Our results confirm that sulfide minerals, locally pyrrhotite in particular, are the dominant source of these metals, with oxidation processes amplified by the internal structure and hydrogeological properties of rock glaciers. As they allow both the storage of water and the supply of oxygen, rock glaciers create conditions that can sustain sulfide oxidation and metal mobilization over extended periods, enriching their spring waters in the released solutes.

As climate change accelerates permafrost ice melt and thereby increases the exposure of sulfide-bearing rocks to oxygen and meltwater infiltration, it likely enhances sulfide oxidation and the associated release of acidity and potentially toxic metals. This has critical consequences for water quality in alpine permafrost regions, where the contamination of freshwater resources with dissolved metals may pose a threat to aquatic ecosystems and complicate the use of these waters for drinking purposes. While the demand for drinking water in these areas grows, particularly due to increased tourism, the high metal concentrations in permafrost-related spring waters can render them unsuitable for direct consumption. Future research should focus on potential mitigation strategies to safeguard water quality in these vulnerable regions.

## Supplementary Material



## Notes

Description of metamorphic evolution of bedrocks at study sites (Supporting Text). Detailed description of spring water isotope analysis (Supporting Text). Detailed description of hydrogeochemical model (Supporting Text). Chemical composition of bedrock minerals at study sites (Supporting Table). Chemical compositions and physical parameters of sampled rock glacier springs (Supporting Table). Statistical comparison of spring water chemistry in this study to a baseline reference data set (Supporting Figure and Table). Sulfate isotope values (sulfur, oxygen) of spring water (Supporting Table). Calculated saturation indices (SI) for rock-forming minerals and possible secondary phases (Supporting Table). Geomorphological maps of the study sites (Supporting Figure). Photographs of mineral coatings observed at the study sites (Supporting Figures). Comparison of Ni-contents in bedrock minerals at study sites (Supporting Figure). Hydrogeochemical model code (PHREEQC Code.pqi, PHREEQC Code (Lazaun).pqi) (ZIP)
