# Supplementary material for: Origin and Evolution of High Nickel Concentrations in Rock Glacier Springs
Source: ACS ES T Water. 2025 Aug 26;5(11):6397–408. doi: 10.1021/acsestwater.5c00542 (PMC12626229; doi:10.1021/acsestwater.5c00542)
Supplement: Supplementary file 1 [file ew5c00542_si_001.zip › Supporting Information.pdf]

The Origin and Evolution of High Nickel Concentrations in Rock Glacier Springs

Simon Seelig, Karl Krainer, Peter Tropper, Michael Pettauer, Albrecht Leis, Thomas Wagner, Giulia Bertolotti, Rudolf Philippitsch, Gerfried Winkler

Supporting Information

This document contains background information on the metamorphic evolution of bedrocks at the study sites, additional information regarding the stable isotope analysis, and full details of the hydrogeochemical model, including the model code which is supplied as two separate files 'PHREEQC Code.pqi' and 'PHREEQC Code (Lazaun).pqi'.

The chemical data of analzyed sulfide, oxide and silicate minerals are given in Supporting Table S1. The chemical compositions and physical parameters of sampled rock glacier springs are reported in Supporting Table S2. The full statistics comparing spring waters in this study to a baseline reference of 195 rock glacier springs from various mountain ranges worldwide are given in Supporting Table S3. Supporting Table S4 provides the isotopic signatures of spring water sulfate and freshwater, as well as the relative contributions of two redox reaction pathways based on oxygen isotopes. Supporting Table S5 summarizes the saturation indices for various mineral phases inferred from the hydrogeochemical model.

Supporting Figure S1 depicts geomorphological maps of the study sites Lazaun, Krummgampen, Wannenkar, and Inneres Hochebenkar. Supporting Figures S2 and S3 show photos of mineral coatings forming whitish crusts at Krummgampen, Supporting Figures S4 and S5 show photos of mineral coatings at Wannenkar. Supporting Figure S6 compares the element contents of nickel between the different mineral groups found in bedrock samples, and Supporting Figure S7 compares the concentrations observed in spring waters of this study to 195 rock glacier springs (baseline reference).

Supporting Information for
The Origin and Evolution of High Nickel Concentrations in Rock Glacier Springs

Content

Supporting Text S1: Metamorphic Evolution of Bedrocks at the Study Sites 3
Supporting Text S2: Spring Water Isotope Analysis..3
Supporting Text S3: Hydrogeochemical Model 5
Supporting Table S1 7
Supporting Table S2 9
Supporting Table S3 9
Supporting Table S4 10
Supporting Table S5 11
Supporting Figure S1 12
Supporting Figure S2 13
Supporting Figure S3 14
Supporting Figure S4 15
Supporting Figure S5 16
Supporting Figure S6 17
Supporting Figure S7 18
References 19

Associated files

PHREEQC Code.pqi

PHREEQC Code (Lazaun).pqi

Supporting Text S1: Metamorphic Evolution of Bedrocks at the Study Sites

The metamorphic host rocks of the Ötztal-Stubai Complex (ÖSC) and their evolution provide the framework to interpret the mineralogical composition and alteration processes observed in the bedrock samples.[1,2] The oldest metamorphic event in the region occurred during the Ordovician (490–460 Ma), resulting in the formation of orthogneisses and scattered migmatites. The Variscan metamorphic overprint spans from 390 to 295 Ma, with the first phase occurring between 373 and 359 Ma, characterized by high-pressure metamorphism that led to the formation of eclogites in the central part of the ÖSC. The eclogite-facies metamorphism is estimated to have occurred at temperatures of 650–750 °C and pressures of 20–28 kbar. The dominant Variscan amphibolite-facies metamorphism took place around 330–350 Ma, with P-T conditions estimated at 570–640 °C and 5.8–7.5 kbar in the northwestern part of the ÖSC. Following this, diabase dikes intruded into the polymetamorphic basement. The youngest metamorphic event in the Austroalpine basement occurred during the Cretaceous Eo-Alpine orogeny (100–73 Ma). The intensity of the Eo-Alpine overprint varies across the ÖSC, increasing from NW (lower greenschist-facies) to SE (epidote-amphibolite facies), reaching temperatures of 550–600 °C and pressures ≥ 11 kbar in the Schneeberg Complex.

Supporting Text S2: Spring Water Isotope Analysis

Spring water sulfate can originate from atmospheric, pedospheric, and lithospheric sources.[3] Its sulfur and oxygen isotope compositions reflect both the isotopic characteristics of the source materials and the fractionation effects of subsequent processes:[4,5] while the distinct $\delta^{18}O_{(SO_4)}$ values preserve source-specific signatures due to the kinetic stability of the sulfate ion and negligible oxygen isotopic exchange with ambient water under low-temperature conditions, the $\delta^{34}S_{(SO_4)}$ values may record evidence of sulfur cycling and mixing processes.

Typical $\delta^{34}S_{(SO_4)}$ values range from -40 ‰ to +40 ‰, with overlapping ranges among individual sources (see Figure 4 of the main document).[3,5,6] Atmospheric $\delta^{34}S_{(SO_4)}$ varies regionally (blue box in Figure 4), mostly ranging between -5 ‰ and +25 ‰, depending on the origin of sulfate: natural sulfur sources, such as sea spray-derived sulfate, dimethylsulfide, terrestrial biogenic emissions, and volcanic emissions, exhibit $\delta^{34}S_{(SO_4)}$ values ranging from approximately 0 ‰ to +21 ‰, whereas anthropogenic sulfate in precipitation typically falls between -3 ‰ and +11 ‰.[3,7-11] In soil waters (green box), the $\delta^{34}S_{(SO_4)}$ is similar to that of atmospheric deposition, since sulfur cycling through sorption or biotic processes hardly affects the isotopic sulfur composition.[5,12] When reduced inorganic sulfur compounds are the primary source of sulfate (yellow box), its isotopic signature closely reflects those of the reduced sulfur source, as sulfur fractionation during sulfide mineral oxidation is minimal under field conditions.[4,13,14] In sedimentary rocks, $\delta^{34}S_{(SO_4)}$ values roughly range from -40 ‰ to +15 ‰, while igneous rocks typically exhibit values between 0 ‰ and +5 ‰, with metamorphic rocks preserving isotopic signatures that reflect their sedimentary or igneous protoliths.[3,15] In contrast, evaporites show high $\delta^{34}S_{(SO_4)}$ values, ranging from +10 ‰ to +35 ‰ (purple box).[15-17]

As complementary tracer, $\delta^{18}O_{(SO_4)}$ in spring water enables the differentiation of individual sulfate sources. Atmospheric sulfate deposition typically ranges from +7 to +17 ‰ in $\delta^{18}O_{(SO_4)}$ (blue box in Figure 4), with sulfates from industrial stacks occasionally exceeding this range (up to +45 ‰).[3,18,19] In contrast to sulfur isotopes, oxygen fractionation is influenced by the cycling of atmospheric sulfate

through organic soils, resulting in a significant depletion in $\delta^{18}O_{(SO_4)}$ values (green box).[5,12] For sulfide oxidation (yellow box), the $\delta^{18}O_{(SO_4)}$ values of sulfate depend primarily on the isotopic signature of the oxygen source involved in sulfide oxidation, which commonly ranges from -25 ‰ to +5 ‰.[4,20-22] Evaporites typically display $\delta^{18}O_{(SO_4)}$ values ranging from +7 ‰ to +20 ‰ (purple box).[3,23]

The isotopic composition of sulfate in the seven rock glacier springs of this study indicates that it primarily originates from the oxidation of reduced inorganic sulfur compounds, such as pyrrhotite or pyrite, which occur as bedrock sulfide minerals (Figure 4, Supporting Table S4). The lack of well-developed soil and the absence of evaporites in the rock glacier catchments further support this interpretation. The dual-isotope approach highlights mineral weathering as the primary source of sulfate, with atmospheric deposition playing a minor or undetectable role, reflected in the low $\delta^{34}S_{(SO_4)}$ and $\delta^{18}O_{(SO_4)}$ values of the rock glacier spring waters.

As outlined above, the $\delta^{18}O_{(SO_4)}$ composition of sulfate in spring water reflects contributions from multiple oxygen-bearing species involved in sulfide oxidation, predominantly atmospheric oxygen and freshwater. The oxidation of sulfide minerals, such as pyrite, might proceed by two reaction pathways that differ regarding the origin of sulfate oxygen, i.e.[13,24,25]

$$FeS_2 + 14\ Fe^{3+} + 8\ H_2O \rightarrow 15\ Fe^{2+} + 2\ SO_4^{2-} + 16\ H^+ \quad (1)$$

$$2\ FeS_2 + 7\ O_2 + 2\ H_2O \rightarrow 2\ Fe^{2+} + 4\ SO_4^{2-} + 4\ H^+ \quad (2)$$

where in reaction (1) the sulfate oxygen is derived exclusively from H_2O while in reaction (2), it is derived from O_2 (87.5 %) and H_2O (12.5 %). The Fe^{3+}-mediated reaction (1) requires ferrous iron (Fe^{2+}) to oxidize to ferric iron (Fe^{3+}), i.e.

$$4\ Fe^{2+} + O_2 + 4\ H^+ \rightarrow 4\ Fe^{3+} + 2\ H_2O \quad (3)$$

The large $\delta^{18}O$ contrast between O_2 (+23.88 ‰)[26] and H_2O (varying from -14.11 ‰ to -13.34 ‰ between the springs, Supporting Table S4) allows us to evaluate the relative participation of the two reaction pathways (1) and (2) using a simple isotope balance equation. Assuming that the stoichiometry of reactions (1) and (2) also governs the isotope composition, and denoting by x the fraction of SO_4^{2-} derived from reaction (1), the $\delta^{18}O_{(SO_4)}$ of sulfate can be expressed as:[24]

$$\delta^{18}O_{(SO_4)} = x\left(\delta^{18}O_{(H_2O)} + \varepsilon_w\right) + (1 - x)\left(0.875\left(\delta^{18}O_{(O_2)} + \varepsilon_a\right) + 0.125\left(\delta^{18}O_{(H_2O)} + \varepsilon_w\right)\right) \quad (4)$$

where $\delta^{18}O_{(SO_4)}$ is the oxygen isotope composition of sulfate (‰), $\delta^{18}O_{(H_2O)}$ is the oxygen isotope composition of spring water (‰), $\delta^{18}O_{(O_2)}$ is the isotope composition of atmospheric oxygen (‰), ε_a accounts for the shift in $\delta^{18}O_{(O_2)}$ values due to kinetic oxygen isotope fractionation during incorporation of atmospheric oxygen into sulfate (-11.2 ‰)[13,24], and ε_w accounts for the shift in $\delta^{18}O_{(H_2O)}$ values due to incorporation of H_2O-bound oxygen into sulfate (+4.1 ‰)[13,24]. The factors reflect the relative contributions of O_2 and H_2O, as indicated in reactions (1) and (2). To constrain the local oxygen isotope composition of subsurface water, we used the long-term mean $\delta^{18}O_{(H_2O)}$ of spring water at each site, assuming this value provides a reasonable approximation of the local subsurface water signature (reported in Supporting Table S4). This approach was applied only at springs with at least one year of water sampling to capture seasonal variability and reliably estimate the subsurface water signal; the individual measurements are publicly available.[27-29]

By solving equation (4) for x, we calculated the relative contribution of Fe^{3+}-mediated sulfide oxidation (reaction (1)) to the oxygen in sulfate, yielding values between 68–75%, as shown in Supporting Table S4. These results are consistent with previous findings indicating that Fe^{3+}-mediated oxidation dominates under acidic conditions, often proceeding quasi-autocatalytically due to the increased solubility and reactivity of Fe^{3+} at low pH.[13,24] Although the reaction may become self-limiting due to constraints on Fe^{3+} regeneration (e.g., limited O_2 availability) or formation of passivating layers such as elemental sulfur[25], the isotopic data support a dominant role of Fe^{3+}-based oxidation. As pH decreases, the dominant oxidation mechanism is thought to shift from O_2-mediated to Fe^{3+}-mediated pathways. Initially, dissolved molecular oxygen plays a crucial role by oxidizing Fe^{2+} to Fe^{3+}, thereby initiating the sulfide oxidation process. Once sufficiently low pH conditions are established – particularly in weakly buffered systems – the increased solubility and reactivity of Fe^{3+} promote a self-sustaining oxidation cycle.[30,31] Ferric iron may then be regenerated either through continued Fe^{2+} oxidation or directly via sulfide dissolution. Under such conditions, sulfate production can proceed even in the absence of sustained molecular oxygen input. However, while our data indicate that Fe^{3+}-mediated pyrite oxidation likely contributes substantially to SO_4^{2-} formation, alternative pathways, such as direct oxidation by atmospheric O_2 or the involvement of reactive oxygen species (ROS), cannot be ruled out, particularly under near-neutral pH conditions.[32]

Supporting Text S3: Hydrogeochemical Model

This document and the corresponding PHREEQC input files (*.pqi) provide the full details of the hydrogeochemical model, including the model code. These are provided in the files ‘PHREEQC Code.pqi’ and ‘PHREEQC Code (Lazaun).pqi’, which contain the full model input.

The computer code PHREEQC V3[33] (Parkhurst & Appelo, 2013) with the *phreeqc.dat* and the *carbfix.dat* database[34] was used to calculate ion balance errors, aqueous speciation and saturation indices (SI = log(IAP/K); IAP: ion activity product; K: solubility product) for primary carbonate, silicate, sulfate, and sulfide minerals (calcite, dolomite, gypsum, anorthite, albite, K-feldspar, quartz, K-mica (muscovite), pyrite, pyrrhotite) and for secondary precipitates, such as kaolinite, the amorphous phases of SiO_2, $Al(OH)_3$ and $Fe(OH)_3$, basaluminite[35-37] and allophanes (short-range ordered Al/Si-phases)[38]. Additionally, the internal partial pressure of CO_2 was calculated to account for gas-water interactions. Supersaturated phases/minerals are not dissolving or may precipitate depending on kinetic constraints (SI > 0), while undersaturated phases (SI < 0), if present, can dissolve but cannot precipitate from the solution.

The inverse modeling code was used to determine the transfer from an initial ‘pure’ water (rain- or meltwater) to the observed composition of rock glacier spring waters through mass balance calculation to simulate Natural Acid Rock Drainage (NARD).[39-41] The initial ‘pure water’ was equilibrated with gaseous CO_2 and O_2 at partial pressures fixed to atmospheric levels (log(pO_2) = -0.7, log(pCO_2) = -3.4). The model consists of three main requirements: i) the given mineral assemblage of the local host rock, ii) the secondary precipitated phases, and iii) the exchange of dissolved CO_2 and O_2. The mineral assemblage was determined by the petrography of the Krummgampen rock glacier host rock by anorthite ($CaAl_2Si_2O_8$) and albite ($Na_2AlSi_3O_8$) as plagioclase solid-solution endmembers, biotite ($KMg_{1.2}Fe_{1.4}Al_{1.8}Si_{2.6}O_{10}(OH)_2$), chlorite ($Mg_3Fe_2Al_2Si_3O_{10}(OH)_8$), and pyrrhotite ($Fe_7S_8$). The stoichiometric reaction equations and mineral composition for anorthite and albite were taken from the *carbfix.dat* database. The compositions for biotite (5), chlorite (6), and pyrrhotite (7) were inferred from microprobe measurements of these minerals in thin sections as follows:

$$KMg_{1.2}Fe_{1.4}Al_{1.8}Si_{2.6}O_{10}(OH)_2 + 4.4\ H^+ + 5.6\ H_2O \leftrightarrow$$
$$\leftrightarrow K^+ + 1.2\ Mg^{2+} + 1.4\ Fe^{2+} + 1.8\ Al(OH)_4^- + 2.6\ H_4SiO_4 \tag{5}$$

$$Mg_3Fe_2Al_2Si_3O_{10}(OH)_8 + 16\ H^+ \leftrightarrow 2\ Al^{3+} + 2\ Fe^{2+} + 6\ H_2O + 3\ H_4SiO_4 + 3\ Mg^{2+} \quad (6)$$

$$Fe_7S_8 + 8\ H^+ + 2\ e^- \leftrightarrow 7\ Fe^{2+} + 8\ HS^- \quad (7)$$

The primary mineral assemblage was set to 'dissolve only'. Secondary phases (e.g. allophane and hisingerite[38] or Fe-(hydr-)oxides) were defined by the amorphous phases of SiO_2, $Al(OH)_3$ and $Fe(OH)_3$ (*carbfix.dat*) and set to 'precipitate only'. Gas exchange with the gas phase was simulated bidirectionally, allowing both uptake and degassing of CO_2 and O_2. Note that the inverse hydrogeochemical model cannot discriminate between the two reaction pathways (1) and (2), which explains the high calculated oxygen demand of up to 479 mg/L (Figure 5 of the main document) far exceeding the solubility of dissolved O_2 (~11.5 mg/L at 1 °C and ~80 kPa at 2000 m a.s.l.). This result reflects the total stoichiometric oxygen demand of the modeled reaction pathway rather than the actual dissolved O_2 available under equilibrium conditions.

The mineral assemblage in the model was simplified using only albite, anorthite, chlorite, biotite, and pyrrhotite to include the minimum necessary phases responsible for providing the major dissolved components like Na^+, K^+, Ca^{2+}, and Mg^{2+}. Other minerals found in the analyzed rocks, such as amphibole, garnet, staurolite, kyanite, or epidote, may also contribute to the major dissolved compounds, depending on their abundance and individual reaction rates. These minerals will also supply Al, Si, and Fe, which are expected to precipitate into secondary phases. However, increasing the number of dissolvable components also increases the number of possible inverse models. By reducing the components of the model, the underlying principal process of NARD was made visible and could be applied to all study sites with reasonable uncertainties.

A third simplification was made by using the amorphous $Fe(OH)_3$, SiO_2, and $Al(OH)_3$ phases as end-members for all major secondary precipitates. These end-members sum up the variety of amorphous or short-range ordered secondary Si/Al/Fe-phases like hydroxy aluminosilicates (HAS), hydrous ferric silicates (HFS), and their solid solutions (HAS-HFS)[38], as well as Fe-(hydr)oxides (iron ochre formation)[42-44], gibbsite, and amorphous silica, which may mature to secondary clay minerals such as kaolinite or montmorillonite. Note that primary carbonates, such as calcite and dolomite, were excluded from the model because of their absence in the host rock. This is also supported by the low pH of the spring waters and negative saturation indices (mean SI = -3.89 for calcite and -7.99 for dolomite, Supporting Table S5). The fast dissolution kinetics of carbonate rocks[45,46] compared to silicate rocks[47] would quickly buffer the pH, bringing SI-values closer to saturation, which was not observed. Quartz was excluded from the model due to its slow reaction kinetics[48], particularly near saturation, as indicated by the calculated SI values (Supporting Table S5). While a more detailed model incorporating trace metals would require extensive, high-resolution datasets, this simplified approach supports a meaningful process-based interpretation that maintains consistency with available observations. This approach does not aim to reproduce all geochemical variability, but rather to constrain the main geochemical pathways driving NARD formation in rock glacier systems.

Supporting Table S1: *Chemical composition of the sulfides, oxides and silicates, given as element wt.% in the sulfides and oxide-wt.% for the oxides and silicates (Fe, Cu, Ni, Zn, Mn, Co, S, P). In contrast, the data in Supporting Figure S6 are given in element wt.% of Ni and Mn for all mineral groups for a direct comparison between them (KG: Krummgampen, WA: Wannenkar, LA: Lazaun, Po: pyrrhotite, Py: pyrite, FeOOH: Fe-hydroxide, Cp: chalcopyrite, Grt: garnet; Ms: muscovite; Bt: biotite; Chl: chlorite; Hbl: hornblende, Ilm: ilmenite, n.d.: not detected).*

Sulfides

Study site	Fe	Cu	Ni	Zn	Mn	Co	S	P	Total	Mineral
KG	59.88	n.d.	0.16	0.01	<0.01	0.13	39.39	n.d.	99.58	Po
KG	59.20	0.06	0.09	n.d.	0.15	0.17	39.12	n.d.	98.80	Po
KG	60.12	n.d.	0.11	0.04	0.17	0.19	39.37	n.d.	99.99	Po
KG	59.20	0.07	0.13	0.02	0.02	0.17	39.86	n.d.	99.48	Po
WA	59.20	0.06	0.10	n.d.	0.15	0.17	39.12	n.d.	98.80	Po
WA	60.12	n.d.	0.11	0.04	0.17	0.19	39.37	n.d.	99.99	Po
KG	59.16	n.d.	1.04	n.d.	n.d.	n.d.	39.79	n.d.	100.00	Po
KG	60.55	n.d.	0.86	n.d.	n.d.	n.d.	38.22	0.27	100.00	Po
KG	58.51	n.d.	0.96	n.d.	n.d.	n.d.	40.53	n.d.	100.00	Po
KG	58.41	n.d.	1.35	n.d.	n.d.	n.d.	40.24	n.d.	100.00	Po
KG	59.29	n.d.	0.52	n.d.	n.d.	n.d.	40.19	n.d.	100.00	Po
KG	58.39	n.d.	0.67	n.d.	n.d.	n.d.	40.94	n.d.	100.00	Po
KG	46.03	0.06	0.17	n.d.	n.d.	0.15	53.59	n.d.	99.99	Py
KG	46.00	n.d.	0.17	n.d.	n.d.	0.11	53.72	n.d.	100.00	Py
KG	45.81	0.01	0.15	0.02	<0.01	0.14	53.86	n.d.	100.00	Py
LA	29.57	33.47	0.02	0.06	0.01	0.05	35.72	n.d.	98.9	Cp

Oxides

Study site	SiO_2	Al_2O_3	TiO_2	NiO	FeO	MnO	CaO	Na_2O	K_2O	Total	Mineral
WA	2.77	0.72	0.08	0.74	72.83	0.04	0.15	0.04	n.d.	77.41	FeOOH
WA	4.04	1.24	0.03	0.77	73.64	0.09	0.19	0.05	n.d.	80.13	FeOOH
WA	4.63	1.75	0.01	1.00	72.39	0.04	0.11	0.07	0.01	80.03	FeOOH
WA	5.48	4.12	0.17	0.06	66.08	0.01	0.11	0.11	0.02	76.18	FeOOH
WA	5.42	2.88	n.d.	0.11	65.67	0.01	0.07	0.21	0.03	74.41	FeOOH
WA	5.65	1.81	n.d.	0.79	71.33	n.d.	0.14	0.05	n.d.	79.78	FeOOH
WA	5.38	0.25	n.d.	0.22	68.23	n.d.	0.18	0.21	0.03	74.49	FeOOH
WA	5.89	2.28	n.d.	0.77	72.48	0.01	0.84	0.06	0.01	82.35	FeOOH
WA	1.62	2.04	0.06	0.20	70.64	n.d.	0.53	0.09	n.d.	75.27	FeOOH
WA	2.23	3.87	0.69	0.02	66.42	n.d.	0.88	0.18	0.02	74.73	FeOOH
WA	5.62	1.90	0.01	0.98	72.10	0.07	0.48	0.01	n.d.	81.28	FeOOH
WA	25.78	15.22	0.09	0.18	37.74	0.04	8.91	2.87	0.07	90.92	FeOOH
LA	0.12	n.d.	52.22	0.04	42.93	1.94	0.18	n.d.	n.d.	97.60	Ilm

Supporting Table S1 (continued): *Chemical composition of the sulfides, oxides and silicates, given as element wt.% in the sulfides and oxide-wt.% for the oxides and silicates (Fe, Cu, Ni, Zn, Mn, Co, S, P). In contrast, the data in Supporting Figure S6 are given in element wt.% of Ni and Mn for all mineral groups for a direct comparison between them (KG: Krummgampen, WA: Wannenkar, LA: Lazaun, Po: pyrrhotite, Py: pyrite, FeOOH: Fe-hydroxide, Cp: chalcopyrite, Grt: garnet; Ms: muscovite; Bt: biotite; Chl: chlorite; Hbl: hornblende, Ilm: ilmenite, n.d.: not detected).*

Silicates – paragneiss

Study site	SiO_2	Al_2O_3	TiO_2	NiO	FeO	MnO	CaO	Na_2O	K_2O	Total	Mineral
KG	37.76	21.34	0.01	0.02	28.26	9.15	2.51	0.03	n.d.	101.34	Grt
KG	46.64	36.25	0.76	0.01	0.77	0.01	n.d.	0.86	8.32	94.16	Ms
KG	34.20	19.09	1.60	0.01	20.52	0.24	n.d.	0.08	8.07	93.55	Bt
KG	26.51	19.86	0.07	n.d.	25.88	0.32	0.01	0.00	0.03	87.63	Chl
KG	26.66	19.33	0.05	n.d.	25.63	0.34	0.02	0.02	0.01	87.00	Chl

Silicates – amphibolite

Study site	SiO_2	Al_2O_3	TiO_2	NiO	FeO	MnO	CaO	Na_2O	K_2O	Total	Mineral
LA	44.40	13.48	0.58	0.04	12.52	0.26	10.68	1.78	0.44	96.53	Hbl
LA	26.70	20.46	0.07	0.05	20.56	0.19	0.07	0.01	0.02	85.97	Chl
LA	46.80	9.69	0.97	0.04	11.91	0.22	11.32	1.43	0.23	96.46	Hbl
LA	45.95	10.78	0.82	0.03	12.13	0.15	11.49	1.45	0.26	96.69	Hbl
LA	26.27	21.19	0.06	0.02	20.52	0.15	0.13	n.d.	0.00	86.34	Chl

Supporting Table S2: *Chemical compositions and physical parameters of sampled rock glacier springs. The table includes electrical conductivity (EC, µS/cm), pH (-), water temperature (WT, °C), oxygen content (O_2, mg/L), oxygen saturation (O_2, %), and the concentrations of major dissolved components (Na, K, Mg, Ca, HCO_3, Cl, SO_4, Si) in mg/L. Trace element concentrations (Al, Co, Cu, Fe, Li, Mn, Ni, Zn) are reported in µg/L.*

**Name**	**Date**	**WT**	**pH**	**EC**	**O_2**	**O_2 sat.**	**Na**	**K**	**Mg**	**Ca**	**HCO_3**	**Cl**	**SO_4**	**Si**	**Al**	**Co**	**Cu**	**Fe**	**Li**	**Mn**	**Ni**	**Zn**
		°C		µS/cm	mg/L	%	mg/L	mg/L	mg/L	mg/L	mg/L	mg/L	mg/L	mg/L	µg/L	µg/L	µg/L	µg/L	µg/L	µg/L	µg/L	µg/L
Hochgurgl (HG)	30.08.2021	2.5	5.02	333	9.7	98.0	1.10	3.12	11.5	37	0.61	0.28	143	4.13	564	19	17	2	11	59	105	91
Inneres Hochebenkar (IH)	30.08.2021	2.1	6.12	171	9.8	98.0	0.81	2.88	5.5	18	1.83	0.12	70	2.58	151	6	5	<1	6	7	38	21
Krummgampen (KG2)	01.09.2021	1.0	4.98	446	10.4	97.0	2.63	1.86	22.7	39	0.61	0.31	204	5.89	2592	37	17	2	8	387	226	158
Krummgampen (KG3)	01.09.2021	0.6	5.20	563	10.1	92.7	2.76	2.11	27.9	52	0.61	0.42	252	6.58	2259	28	30	<1	10	284	265	162
Krummgampen (KG4)	01.09.2021	1.0	5.20	527	10.4	96.5	2.57	1.87	26.5	51	0.61	0.42	246	5.92	2330	40	32	1	15	445	253	148
Lazaun (LA_{Jan})	01.01.2007	1.5*	6.91	220	-	-	0.37	1.08	4.8	30	-	0.23	82	0.95	< 1	-	-	-	< 1	-	-	-
Lazaun (LA_{Aug})	26.08.2007	1.9	6.61	239	-	-	0.97	1.67	5.6	33	-	0.17	117	1.04	5	-	-	-	1	-	-	-
Lazaun (LA_{Oct})	16.10.2007	1.5	7.17	249	-	-	0.40	1.26	6.3	37	-	0.18	121	2.30	9	-	-	-	< 1	-	-	-
Wannenkar (WA)	31.08.2021	1.3	6.65	1188	10.1	97.0	3.17	1.07	81.1	132	9.15	1.24	637	4.45	219	23	7	<1	18	67	295	140

*WT was estimated for thermodynamic calculations.

Supporting Table S3: *Statistical comparison of spring water chemistry in this study to a baseline reference dataset of 195 rock glacier springs[49]. The number of springs analyzed varies by component, as not all components were measured at every spring in either this study or the baseline reference. The one-sided rank sum test was used to assess whether the springs in this study exhibit elevated concentrations.[50,51] Reported statistics include the test statistic (W), p-value, and the magnitude of differences between the two groups, represented by the Hodges-Lehmann estimator (Δ), which corresponds to the median of all pairwise differences between measurements in the two groups.[52,53]*

	**Na**	**K**	**Mg**	**Ca**	**Cl**	**HCO_3**	**SO_4**	**Si**	**Al**	**Cu**	**Fe**	**Mn**	**Ni**	**Zn**
**Number of springs (this study)**	7	7	7	7	7	6	7	7	7	6	6	6	6	6
**Number of springs (baseline reference)**	195	195	195	195	195	195	195	93	186	186	188	184	188	186
**Test statistic (W)**	1155	1534	1504	1498	785	83	1575	469	1357	1060	565	1042	1090	1077
**p-value**	6.3×10^{-2}	1.1×10^{-4}	2.1×10^{-4}	2.4×10^{-4}	7.1×10^{-1}	1.0×10^{-0}	2.8×10^{-5}	2.8×10^{-1}	5.9×10^{-4}	3.6×10^{-5}	5.0×10^{-1}	1.1×10^{-5}	2.3×10^{-5}	4.2×10^{-5}
**Hodges-Lehmann estimator (Δ)**	0.4 mg/L	1.0 mg/L	6.5 mg/L	25.4 mg/L	-0.1 mg/L	-14.8 mg/L	116.8 mg/L	0.4 mg/L	214 µg/L	17 µg/L	0 µg/L	66 µg/L	226 µg/L	139 µg/L

Supporting Table S4: *$\delta^{34}S_{(SO_4)}$ and $\delta^{18}O_{(SO_4)}$ values of spring water sampled at seven springs across five rock glaciers. Spring KG3 was sampled twice, once in June and again in September. $\delta^{18}O_{(H_2O)}$ values represent long-term averages of spring water oxygen sampled under varying hydrologic conditions.[27-29] $\delta^{34}S_{(SO_4)}$ values are expressed relative to the Vienna Cañon Diablo Troilite (VCDT), while $\delta^{18}O_{(SO_4)}$ and $\delta^{18}O_{(H_2O)}$ values are expressed relative to the Vienna Standard Mean Ocean Water (VSMOW).[54,55]*

**Name**	**Date**	**$\delta^{34}S_{(SO_4)}$ VCDT [‰]**	**$\delta^{18}O_{(SO_4)}$ VSMOW [‰]**	**$\delta^{18}O_{(H_2O)}$ VSMOW [‰]**	**Fe^{3+}-mediated oxidation [%]**
Hochgurgl (HG)	30.08.2021	0.6 ± 0.1	-5.9 ± 0.1		
Inneres Hochebenkar (IH)	30.08.2021	-6.0 ± 0.0	-2.8 ± 0.3		
Krummgampen (KG2)	01.09.2021	3.3 ± 0.1	-4.1 ± 0.0	-13.47	73
Krummgampen (KG3)	29.06.2021	2.9 ± 0.2	-4.5 ± 0.2	-13.51	75
Krummgampen (KG3)	01.09.2021	2.8 ± 0.0	-4.3 ± 0.0	-13.51	74
Krummgampen (KG4)	01.09.2021	3.0 ± 0.0	-4.3 ± 0.1	-13.34	74
Lazaun (LA)	30.06.2021	5.2 ± 0.3	-3.6 ± 0.1	-14.11	68
Wannenkar (WA)	31.08.2021	1.3 ± 0.0	-3.9 ± 0.5		

Supporting Table S5: *Calculated saturation indices (SI) for rock-forming minerals and possible secondary phases that may form through weathering processes at the seven rock glacier springs. Potential rock-forming minerals considered by the model include carbonates, sulfates, silicates, and sulfides. Supersaturated phases/minerals are not dissolving and may precipitate depending on kinetic constraints (SI > 0), while undersaturated phases, if present, can dissolve but cannot precipitate from the solution (SI < 0). Note that for Lazaun, three different times (Jan, Aug, Oct) of sampling are given for a single spring.*

		**Carbonates**		**Sulfates**	**Silicates**					**Sulfides**		**Secondary phases**							
**Name**	**charge balance (%)**	**Calcite**	**Dolomite**	**Gypsum**	**Anorthite**	**Albite**	**K-felspar**	**Quartz**	**Muscovite**	**Pyrite**	**Pyrrhotite**	**Kaolinite**	**$SiO_{2(am)}$**	**$Al(OH)_{3(am)}$**	**$Fe(OH)_{3(am)}$**	**Allophane (Al/Si: 1.26)**	**Allophane (Al/Si: 1.64)**	**Allophane (Al/Si: 2.02)**	**Basaluminite**
Hochgurgl (HG)	+0.1	-6.93	-14.10	-1.59	-12.12	-7.79	-4.70	0.18	1.24	-255	-159	1.53	-1.25	-2.69	-0.70	-0.40	-0.60	-1.12	-2.73
Inneres Hoch-ebenkar (IH)	+0.1	-4.66	-9.56	-2.11	-5.83	-5.02	-1.83	-0.02	8.90	-259	-160	5.92	-1.45	-0.29	0.14	4.07	3.93	3.46	4.87
Krummgampen (KG2)	+0.2	-7.06	-14.08	-1.47	-11.28	-6.60	-4.09	0.36	2.59	-256	-159	2.62	-1.08	-2.33	-0.68	0.58	0.30	-0.26	-1.30
Krummgampen (KG3)	+0.6	-6.53	-13.06	-1.29	-9.66	-5.70	-3.16	0.41	4.53	-257	-160	3.74	-1.03	-1.83	-0.72	1.66	1.37	0.79	0.39
Krummgampen (KG4)	+0.1	-6.53	-13.07	-1.31	-9.64	-5.83	-3.31	0.36	4.46	-257	-160	3.73	-1.08	-1.78	-0.74	1.68	1.41	0.85	0.61
Lazaun (LA_{Jan})	+3.8	-2.75	-6.04	-1.85	-8.39	-7.58	-4.46	-0.44	2.85	-263	-162	1.65	-1.88	-2.01	0.56	-0.05	-0.03	-0.41	-3.26
Lazaun (LA_{Aug})	-6.2	-3.32	-7.15	-1.68	-7.05	-6.45	-3.57	-0.41	5.53	-261	-161	3.51	-1.84	-1.11	0.60	1.81	1.82	1.43	0.97
Lazaun (LA_{Oct})	-5.4	-2.17	-4.83	-1.63	-4.76	-5.00	-1.86	-0.06	7.72	-263	-162	4.68	-1.50	-0.88	0.74	2.83	2.71	2.24	0.98
Wannenkar (WA)	+0.2	-2.51	-4.94	-0.70	-2.14	-2.43	-0.25	0.23	12.04	-260	-161	7.99	-1.20	0.48	0.64	6.00	5.77	5.24	7.64

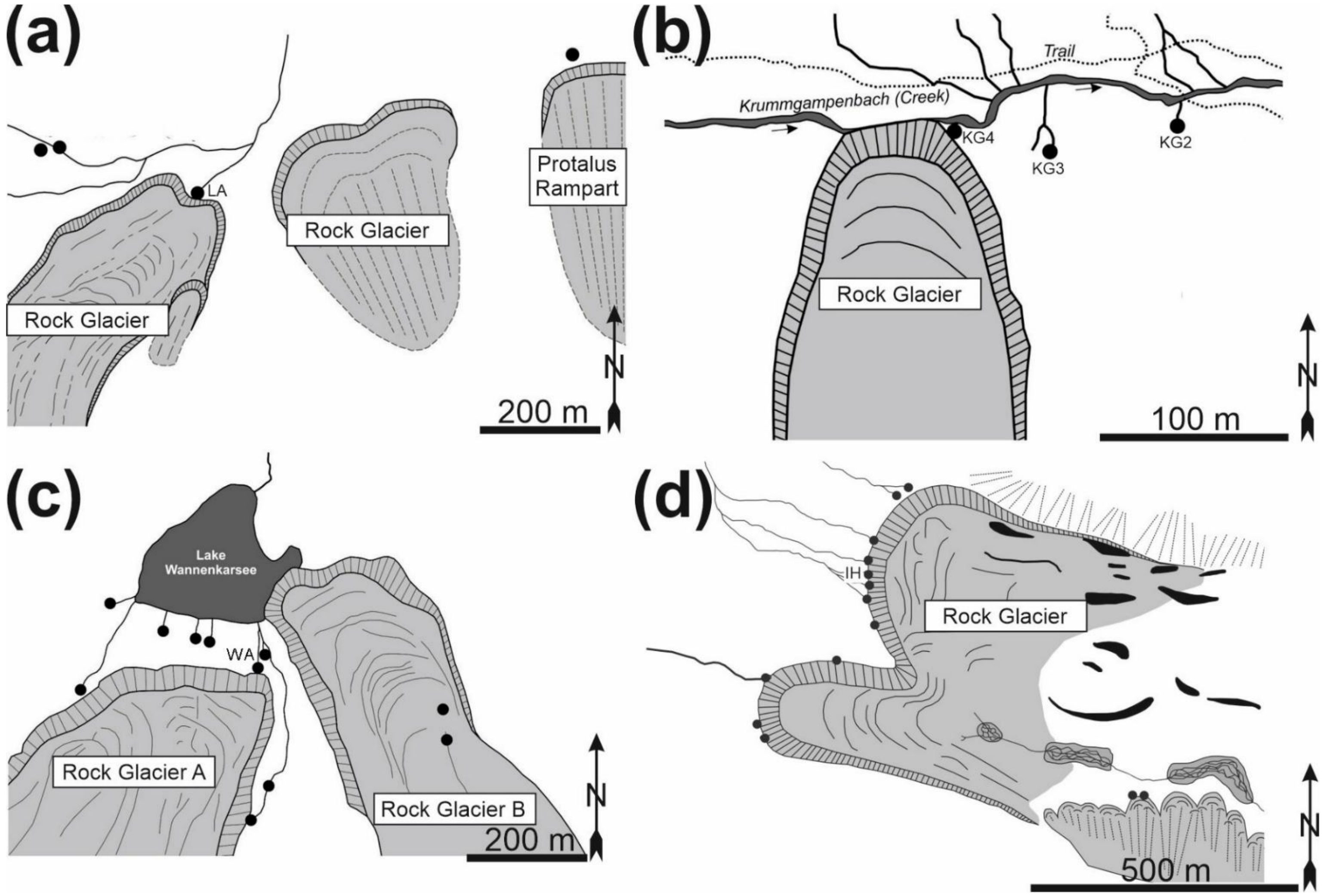

Supporting Figure S1: *Geomorphological maps of the study sites: (a) Lazaun, (b) Krummgampen, (c) Wannenkar, and (d) Inneres Hochebenkar. Rock glaciers are shaded in grey, springs are marked as black dots, rivers are represented by black lines, and lakes are depicted in dark grey. Sampled springs are labelled. Adapted from Krainer et al.*[56-58]

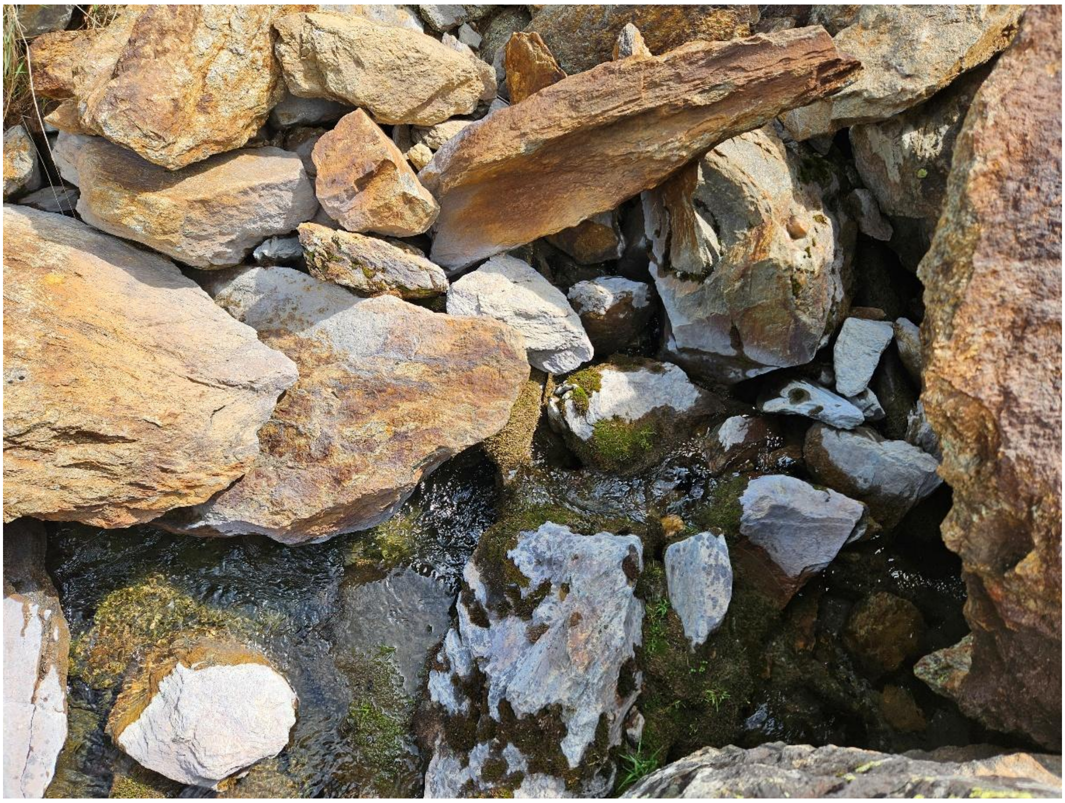

Supporting Figure S2: *Whitish crusts formed by mineral coatings rich in arsenic, copper, nickel, uranium, and yttrium found at Krummgampen rock glacier, after the confluence of rock glacier spring waters with the Krummgampen creek. Detailed chemical and mineralogical analyses are provided by Thies et al.*[59] *Photo: Gerfried Winkler.*

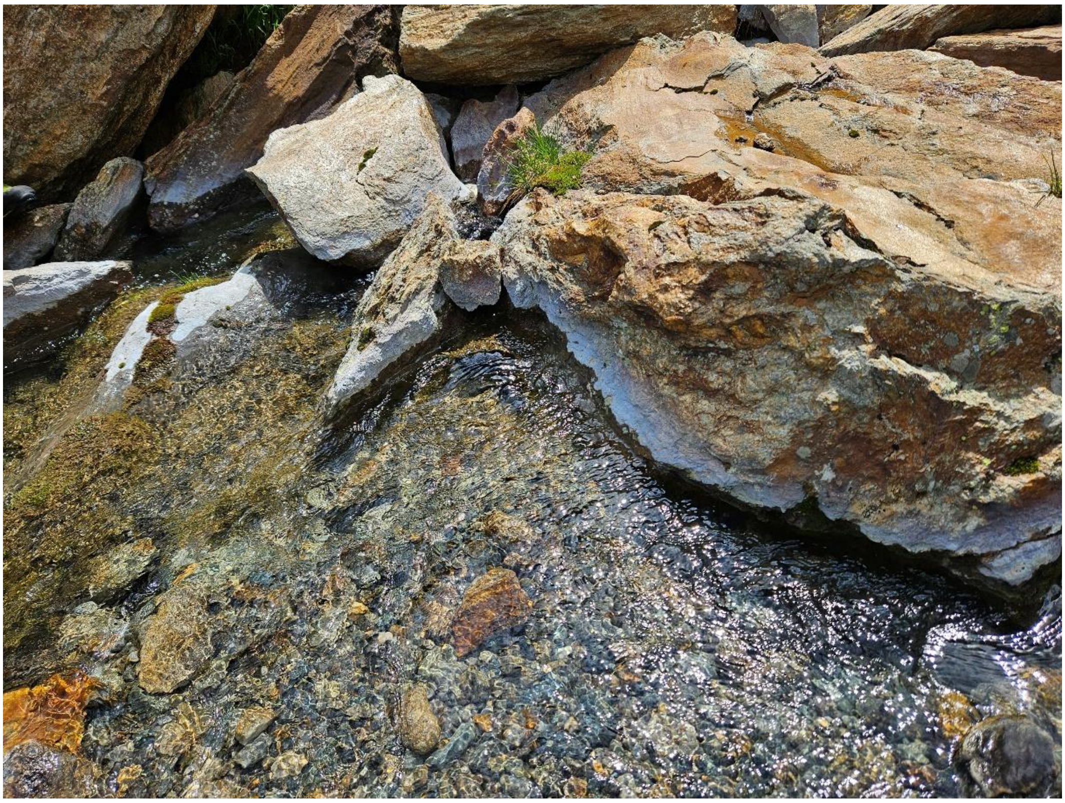

Supporting Figure S3: *Whitish crusts formed by mineral coatings at Krummgampen rock glacier, restricted to the water level range found at the confluence. Chemical and mineralogical analyses are provided by Thies et al.*[59] *Photo: Gerfried Winkler.*

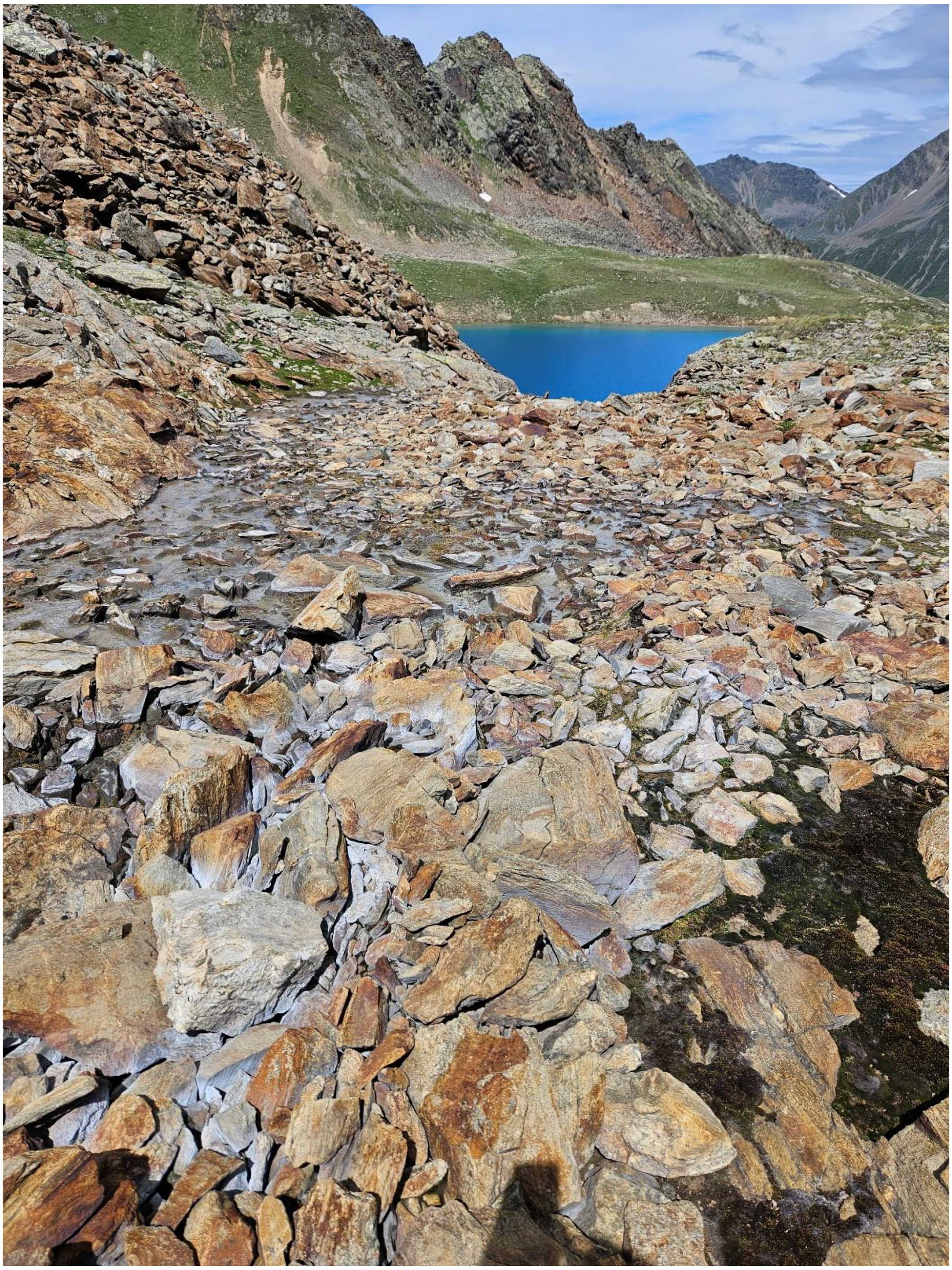

Supporting Figure S4: *Whitish crusts formed by mineral coatings at Wannenkar rock glaciers above Wannenkarsee, downstream from the rock glacier spring. The coatings reflect the range of water levels found near the rock glacier spring. Photo: Gerfried Winkler.*

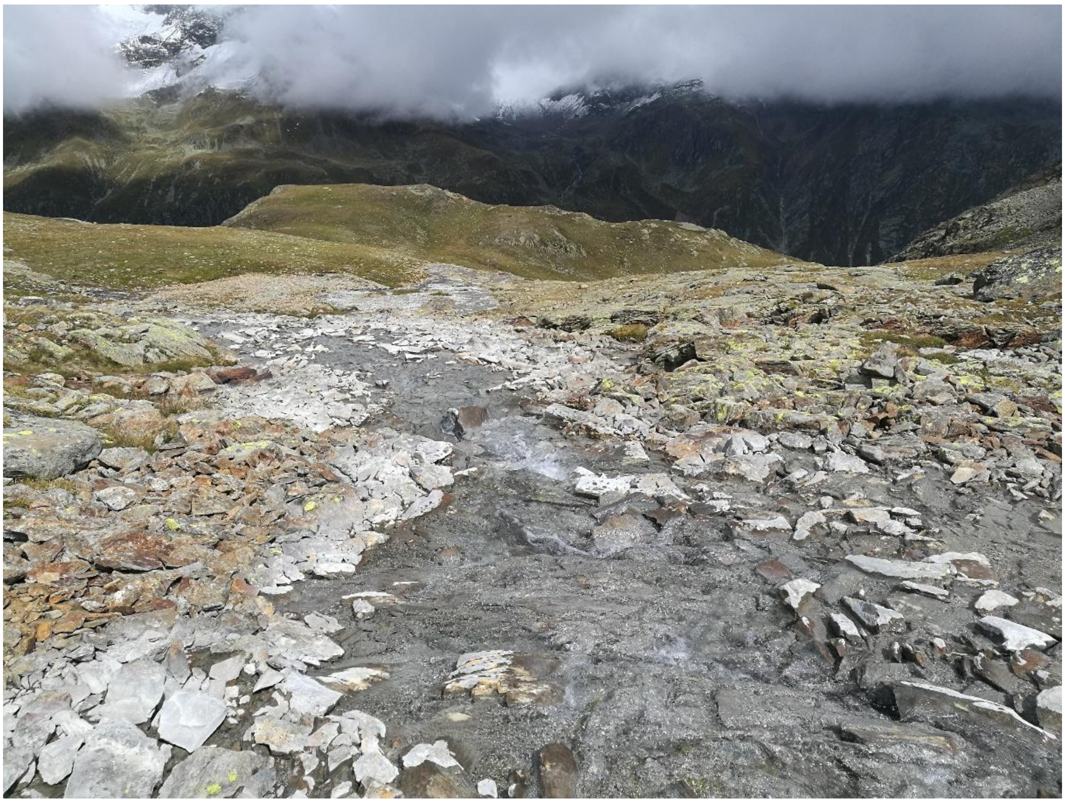

Supporting Figure S5: *Whitish crusts formed by mineral coatings on boulder creeks below Lake Wannenkarsee. The creek draining the lake reflects the chemical composition of the lake, which is governed by Natural Acid Rock Drainage of two rock glaciers above. Photo: Gerfried Winkler.*

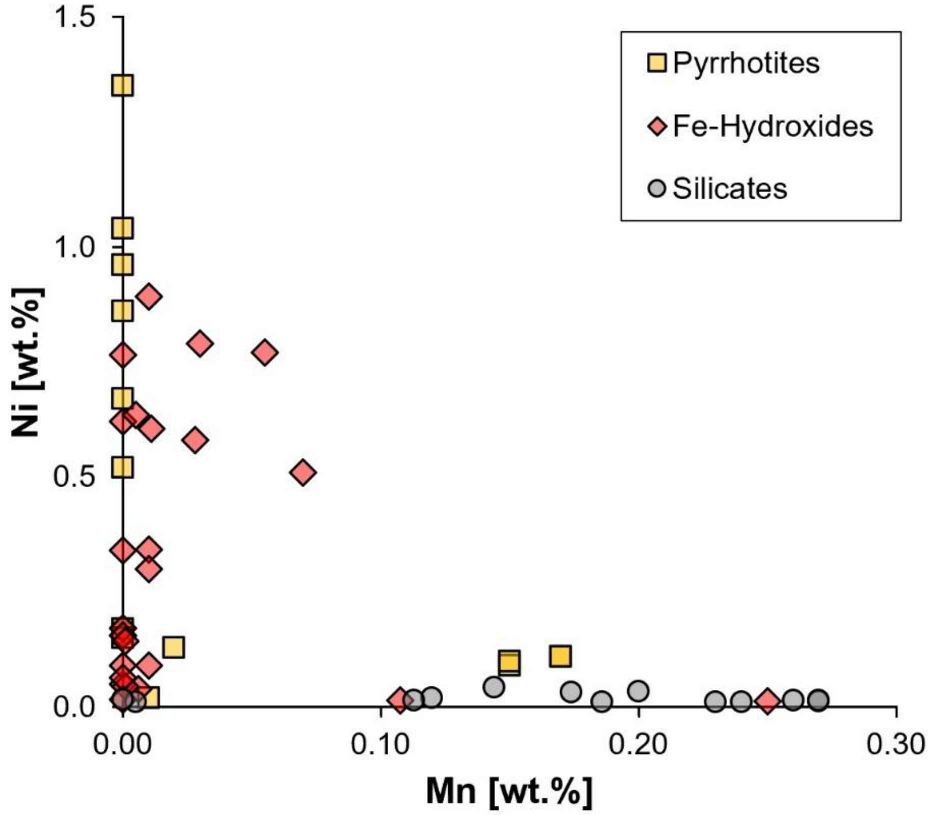

Supporting Figure S6: *Element contents (wt.%) of nickel (Ni) vs. manganese (Mn). Pyrrhotite and Fe-hydroxides contain a large range of Ni contents, while silicate minerals contain the highest Mn and lowest Ni contents. For a direct comparison between the different mineral groups, Ni and Mn contents are reported as element wt.% for all mineral groups.*

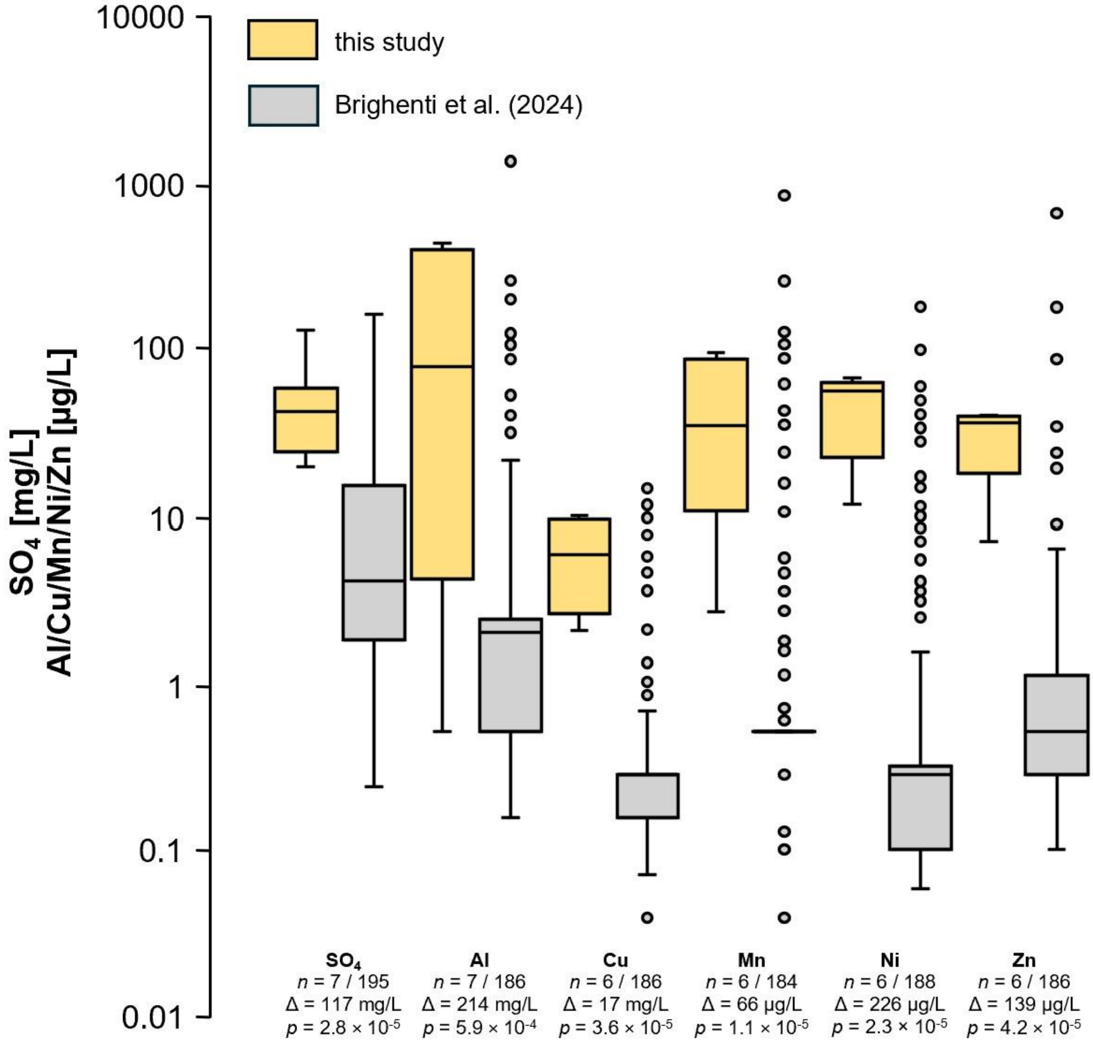

Supporting Figure S7: *Sulfate (SO_4^{2-}), aluminum (Al), copper (Cu), manganese (Mn), nickel (Ni), and zinc (Zn) concentrations observed in spring waters of this study, compared to 195 rock glacier springs from various mountain ranges worldwide, serving as a baseline reference.[49] n indicates the number of springs analyzed in this study and in the baseline dataset, respectively. Δ represents the Hodges-Lehmann estimator, quantifying the difference between this study and the baseline reference.[52,53] The p-value reflects the statistical significance of these differences, determined using the one-sided rank-sum test.[50,51] Note that the number of springs analyzed varies by parameter due to differences in elemental analysis across sites. The full results for all analyzed chemical components are presented in Supporting Table S3.*

References

(1) Hoinkes, G.; Thöni, M. (1993): Evolution of the Ötztal-Stubai, Scarl-Campo and Ulten Basement Units. In: Raumer, J. F.; Neubauer, F. (Ed.): Pre-Mesozoic Geology in the Alps. Springer Berlin Heidelberg, Berlin, Heidelberg, 485–494.

(2) Hoinkes, G.; Krainer, K.; Tropper, P. (2021): Ötztaler Alpen, Stubaier Alpen und Texelgruppe, Stuttgart, Gebr. Borntraeger (Sammlung geologischer Führer, 112).

(3) Krouse, H. R.; Mayer, B. (2000): Sulphur and oxygen isotopes in sulphate. In: Cook, P. G.; Herczeg, A. L. (Ed.): Environmental Tracers in Subsurface Hydrology. Springer US, Boston, MA, 195–231.

(4) Toran, L.; Harris, R. F. (1989): Interpretation of sulfur and oxygen isotopes in biological and abiological sulfide oxidation. Geochimica et Cosmochimica Acta 53 (9), 2341–2348. https://doi.org/10.1016/0016-7037(89)90356-6.

(5) Mitchell, M. J.; Krouse, H. R.; Mayer, B.; Stam, A. C.; Zhang, Y. (1998): Use of Stable Isotopes in Evaluating Sulfur Biogeochemistry of Forest Ecosystems. In: Kendall, C.; McDonnell, J. J. (Ed.): Isotope Tracers in Catchment Hydrology. Elsevier, Amsterdam, 489–518.

(6) Puig, R.; Folch, A.; Menció, A.; Soler, A.; Mas-Pla, J. (2013): Multi-isotopic study (15N, 34S, 18O, 13C) to identify processes affecting nitrate and sulfate in response to local and regional groundwater mixing in a large-scale flow system. Applied Geochemistry 32, 129–141. https://doi.org/10.1016/j.apgeochem.2012.10.014.

(7) Mayer, B. (1998): Potential and limitations of using sulfur isotope abundance ratios as an indicator for natural and anthropogenic induced environmental change. In: International Atomic Energy Agency (IAEA) (Ed.): Isotope Techniques in the Study of Past and Current Environmental Changes in the Hydrosphere and the Atmosphere. Proceedings of a Symposium, Vienna, 14-18 April 1997, Vienna, 423–435.

(8) Norman, A. L.; Barrie, L. A.; Toom-Sauntry, D.; Sirois, A.; Krouse, H. R.; Li, S. M.; Sharma, S. (1999): Sources of aerosol sulphate at Alert. Apportionment using stable isotopes. J. Geophys. Res. 104 (D9), 11619–11631. https://doi.org/10.1029/1999JD900078.

(9) Novák, M.; Kirchner, J. W.; Groscheová, H.; Havel, M.; Černý, J.; Krejčí, R.; Buzek, F. (2000): Sulfur isotope dynamics in two central european watersheds affected by high atmospheric deposition of SOx. Geochimica et Cosmochimica Acta 64 (3), 367–383. https://doi.org/10.1016/S0016-7037(99)00298-7.

(10) Novák, M.; Jacková, I.; Prechová, E. (2001): Temporal trends in the isotope signature of air-borne sulfur in Central Europe. Environ. Sci. Technol. 35 (2), 255–260. https://doi.org/10.1021/es0000753.

(11) Amrani, A.; Said-Ahmad, W.; Shaked, Y.; Kiene, R. P. (2013): Sulfur isotope homogeneity of oceanic DMSP and DMS. Proceedings of the National Academy of Sciences of the United States of America 110 (46), 18413–18418. https://doi.org/10.1073/pnas.1312956110.

(12) Mayer, B.; Feger, K. H.; Giesemann, A.; Jäger, H.-J. (1995): Interpretation of sulfur cycling in two catchments in the Black Forest (Germany) using stable sulfur and oxygen isotope data. Biogeochemistry 30 (1), 31–58. https://doi.org/10.1007/BF02181039.

(13) Taylor, B. E.; Wheeler, M. C.; Nordstrom, D. K. (1984a): Isotope composition of sulphate in acid mine drainage as measure of bacterial oxidation. Nature 308 (5959), 538–541. https://doi.org/10.1038/308538a0.

(14) Taylor, B. E.; Wheeler, M. C.; Nordstrom, D. K. (1984b): Stable isotope geochemistry of acid mine drainage. Experimental oxidation of pyrite. Geochimica et Cosmochimica Acta 48 (12), 2669–2678. https://doi.org/10.1016/0016-7037(84)90315-6.

(15) Strauss, H. (1997): The isotopic composition of sedimentary sulfur through time. Palaeogeography, Palaeoclimatology, Palaeoecology 132 (1-4), 97–118. https://doi.org/10.1016/S0031-0182(97)00067-9.

(16) Strauss, H. (1993): The sulfur isotopic record of Precambrian sulfates. New data and a critical evaluation of the existing record. Precambrian Research 63 (3-4), 225–246. https://doi.org/10.1016/0301-9268(93)90035-Z.

(17) Tostevin, R.; Turchyn, A. V.; Farquhar, J.; Johnston, D. T.; Eldridge, D. L.; Bishop, J. K.B.; McIlvin, M. (2014): Multiple sulfur isotope constraints on the modern sulfur cycle. Earth and Planetary Science Letters 396 (7), 14–21. https://doi.org/10.1016/j.epsl.2014.03.057.

(18) Jamieson, R. E.; Wadleigh, M. A. (1999): A Study of the Oxygen Isotopic Composition of Precipitation Sulphate in Eastern Newfoundland. Water, Air, and Soil Pollution 110 (3/4), 405–420. https://10.1023/A:1005002026009.

(19) Novák, M.; Vile, M. A.; Bottrell, S. H.; Štěpánová, M.; Jačková, I.; Buzek, F.; Přechová, E.; Newton, R. J. (2005): Isotope Systematics of Sulfate-oxygen and Sulfate-sulfur in Six European Peatlands. Biogeochemistry 76 (2), 187–213. https://doi.org/10.1007/s10533-005-4433-7.

(20) Balci, N.; Shanks, W. C.; Mayer, B.; Mandernack, K. W. (2007): Oxygen and sulfur isotope systematics of sulfate produced by bacterial and abiotic oxidation of pyrite. Geochimica et Cosmochimica Acta 71 (15), 3796–3811. https://doi.org/10.1016/j.gca.2007.04.017.

(21) Heidel, C.; Tichomirowa, M. (2011): The isotopic composition of sulfate from anaerobic and low oxygen pyrite oxidation experiments with ferric iron — New insights into oxidation mechanisms. Chemical Geology 281 (3-4), 305–316. https://doi.org/10.1016/j.chemgeo.2010.12.017.

(22) Jasechko, S. (2019): Global Isotope Hydrogeology―Review. Rev. Geophys. 57 (3), 835–965. https://doi.org/10.1029/2018RG000627.

(23) Claypool, G. E.; Holser, W. T.; Kaplan, I. R.; Sakai, H.; Zak, I. (1980): The age curves of sulfur and oxygen isotopes in marine sulfate and their mutual interpretation. Chemical Geology 28 (84), 199–260. https://10.1016/0009-2541(80)90047-9.

(24) van Everdingen, R. O.; Krouse, H. R. (1985): Isotope composition of sulphates generated by bacterial and abiological oxidation. Nature 315, 395–396. https://doi.org/10.1038/315395a0.

(25) Rimstidt, J.D.; Vaughan, D. J. (2003): Pyrite oxidation. A state-of-the-art assessment of the reaction mechanism. Geochimica et Cosmochimica Acta 67 (5), 873–880. https://doi.org/10.1016/S0016-7037(02)01165-1.

(26) Luz, B.; Barkan, E. (2011): The isotopic composition of atmospheric oxygen. Global Biogeochem. Cycles 25 (3), GB3001. https://doi.org/10.1029/2010GB003883.

(27) Wagner, T.; Kainz, S.; Wedenig, M.; Pleschberger, R.; Krainer, K.; Kellerer-Pirklbauer, A.; Ribis, M.; Hergarten, S.; Winkler, G. (2019): Wasserwirtschaftliche Aspekte von Blockgletschern in Kristallingebieten der Ostalpen. Speicherverhalten, Abflussdynamik und Hydrochemie mit Schwerpunkt Schwermetallbelastungen. Hg. v. Bundesministerium für Nachhaltigkeit und Tourismus (BMNT), Wien.

(28) Bertolotti, G.; Krainer, K. (2025): Hydrological data of the Rock Glacier Lazaun stream (South Tyrol, Italy) 2011 et seq. PANGAEA, https://doi.pangaea.de/10.1594/PANGAEA.979976.

(29) Austrian Federal Ministry of Agriculture and Forestry, Climate and Environmental Protection, Regions and Water Management (2025): Water Isotope Map of Austria. Water Information System Austria (WISA). Available from https://maps.wisa.bmluk.gv.at/gewaesserbewirtschaftungsplan-2021?g_bbox=977650,5824234,2010467,6295086&g_card=ngp21_g_wasserisotope. Last access: 16 July 2025.

(30) Paschka, M. G.; Dzombak, D. A. (2004): Use of dissolved sulfur species to measure pyrite dissolution in water at pH 3 and 6. Environmental engineering science, 21(4), 411–420. https://doi.org/10.1089/1092875041358502

(31) Qiu, G.; Luo, Y.; Chen, C.; Lv, Q.; Tan, W.; Liu, F.; Liu, C. (2016): Influence factors for the oxidation of pyrite by oxygen and birnessite in aqueous systems. Journal of Environmental Sciences, 45, 164–176. http://dx.doi.org/10.1016/j.jes.2016.01.012

(32) Hemingway, J. D.; Olson, H.; Turchyn, A. V.; Tipper, E. T.; Bickle, M. J.; Johnston, D. T. (2020): Triple oxygen isotope insight into terrestrial pyrite oxidation. P. Natl. Acad. Sci. 117(14), 7650-7657. doi.org/doi:10.1073/pnas.1917518117.

(33) Parkhurst, D. L.; Appelo, C. A. J. (2013): Description of input and examples for PHREEQC version 3—a computer program for speciation, batch-reaction, one-dimensional transport, and inverse geochemical calculations. US geological survey techniques and methods, 6(A43), 497. https://pubs.usgs.gov/tm/06/a43/

(34) Voigt, M.; Marieni, C.; Clark, D. E.; Gíslason, S. R.; Oelkers, E. H. (2018): Evaluation and refinement of thermodynamic databases for mineral carbonation. Energy Procedia, 146, 81–91. https://doi.org/10.1016/j.egypro.2018.07.012

(35) Carrero, S.; Fernandez-Martinez, A.; Pérez-López, R.; Nieto, J. M. (2017): Basaluminite structure and its environmental implications. Procedia Earth and Planetary Science, 17, 237–240. https://doi.org/10.1016/j.proeps.2016.12.080

(36) Wanner, C.; Pöthig, R.; Carrero, S.; Fernandez-Martinez, A.; Jäger, C.; Furrer, G. (2018): Natural occurrence of nanocrystalline Al-hydroxysulfates: Insights on formation, Al solubility control and As retention. Geochimica et cosmochimica acta, 238, 252–269. https://doi.org/10.1016/j.gca.2018.06.031

(37) Lozano, A.; Fernandez-Martínez, A.; Ayora, C.; Poulain, A. (2018): Local structure and ageing of basaluminite at different pH values and sulphate concentrations. Chem. Geol. 496, 25–33. https://doi.org/10.1016/j.chemgeo.2018.08.002

(38) Baldermann, A.; Stamm, F. M.; Farkaš, J.; Löhr, S.; Ratz, B.; Letofsky-Papst, I.; Dietzel, M. (2024): Precipitation of short-range order hydroxy aluminosilicate (HAS) and hydrous ferric silicate (HFS) at ambient temperature: Insights into mineral formation pathways, crystal chemistry and solubility-stability relationships. Chemical Geology, 646, 121911. https://doi.org/10.1016/j.chemgeo.2023.121911.

(39) Kwong, Y. J.; Whitley, G.; Roach, P. (2009): Natural acid rock drainage associated with black shale in the Yukon Territory, Canada. Applied Geochemistry, 24(2), 221–231. https://doi.org/10.1016/j.apgeochem.2008.11.017

(40) Dold, B. (2017): Acid rock drainage prediction: A critical review. Journal of Geochemical Exploration, 172, 120–132. http://dx.doi.org/10.1016/j.gexplo.2016.09.014

(41) Zarroca, M.; Roqué, C.; Linares, R.; Salminci, J. G.; Gutiérrez, F. (2021): Natural acid rock drainage in alpine catchments: A side effect of climate warming. Science of the total environment, 778, 146070. https://doi.org/10.1016/j.scitotenv.2021.146070.

(42) Jambor, J. L.; Dutrizac, J. E. (1998): Occurrence and constitution of natural and synthetic ferrihydrite, a widespread iron oxyhydroxide. Chemical reviews, 98(7), 2549–2586. https://doi.org/10.1021/cr970105t

(43) Søgaard, E. G.; Aruna, R.; Abraham-Peskir, J.; Koch, C. B. (2001): Conditions for biological precipitation of iron by Gallionella ferruginea in a slightly polluted ground water. Applied Geochemistry, 16(9-10), 1129–1137. https://doi.org/10.1016/S0883-2927(01)00014-2

(44) Sharma, S. K.; Petrusevski, B.; Schippers, J. C. (2005): Biological iron removal from groundwater: a review. Journal of Water Supply: Research and Technology—AQUA, 54(4), 239–247. https://doi.org/10.2166/aqua.2005.0022

(45) Plummer, L. N.; Wigley, T. M. L.; Parkhurst, D. L. (1978): The kinetics of calcite dissolution in co 2 -water systems at 5 degrees to 60 degrees c and 0.0 to 1.0 atm co 2. American Journal of Science, 278(2), 179–216. https://doi.org/10.2475/ajs.278.2.179

(46) Plummer, L. N.; Busenberg, E. (1982): The kinetics of dissolution of dolomite in CO2-H2O systems at 1.5 to 65 degrees C and 0 to 1 atm pCO2, evaluation of the aqueous model for the system CaCO3-CO2-H2O. Geochimica et Cosmochimica Acta, 46, 1–2. https://doi.org/10.2475/ajs.282.1.45

(47) Blum, A. E.; Stillings, L. L. (1995): Feldspar dissolution kinetics. Reviews in mineralogy, 31, 291–352. https://doi.org/10.1515/9781501509650-009

(48) Brady, P. V.; Walther, J. V. (1990): Kinetics of quartz dissolution at low temperatures. Chemical geology, 82, 253–264. https://doi.org/10.1016/0009-2541(90)90084-K

(49) Brighenti, S.; Colombo, N.; Wagner, T.; Pettauer, M.; Guyennon, N.; Krainer, K.; Tolotti, M.; Rogora, M.; Paro, L.; Steingruber, S. M.; Del Siro, C.; Scapozza, C.; Sileo, N. R.; Villarroel, C. D.; Hayashi, M.; Munroe, J.; Liaudat, D. T.; Cerasino, L.; Tirler, W.; Comiti, F.; Freppaz, M.; Salerno, F.; Litaor, M. I.; Cremonese, E.; Di Cella, U. M.; Winkler, G. (2024): Factors controlling the water quality of rock glacier springs in European and American mountain ranges. Science of The Total Environment, 953, 175706. https://doi.org/10.1016/j.scitotenv.2024.175706

(50) Wilcoxon, F. (1945): Individual Comparisons by Ranking Methods. Biometrics Bulletin 1 (6), 80–83. https://doi.org/10.2307/3001968.

(51) Helsel, D. R.; Hirsch, R. M.; Ryberg, K. R.; Archfield, S. A.; Gilroy, E. J. (2020): Statistical Methods in Water Resources, U. S. Geological Survey (USGS) (U. S. Geol. Surv. Techniques and Methods, book 4, chapter A3).

(52) Hodges, J. L.; Lehmann, E. L. (1963): Estimates of Location Based on Rank Tests. The Annals of Mathematical Statistics 34 (2), 598–611. https://doi.org/10.1214/aoms/1177704172.

(53) Hollander, M.; Wolfe, D. A.; Chicken, E. (2014): Nonparametric statistical methods. 3. Ed., Hoboken, New Jersey, John Wiley & Sons Inc (Wiley series in probability and statistics). https://doi.org/10.1002/9781119196037.

(54) Coplen, T. B.; Krouse, H. R. (1998): Sulphur isotope data consistency improved. Nature 392 (6671), 32. https://doi.org/10.1038/32080.

(55) Coplen, T. B.; Bièvre, P. de; Krouse, H. R.; Vocke, R. D.; Gröning, M.; Rozanski, K. (1996): Ratios for light-element isotopes standardized for better interlaboratory comparison. Eos Trans. AGU 77 (27), 255. https://doi.org/10.1029/96EO00182.

(56) Krainer, K.; Ausserer, P.; Bressan, D.; Lang, K.; Mair, V.; Mussner, L.; Nickus, U.; Schmidt, V.; Schiestl, E.-M.; Tessadri, R.; Thies, H.; Tonidandel, D. (2015a): Aufbau und Dynamik ausgewählter Blockgletscher in Nord- und Südtirol. Geo.Alp 12, 75–134.

(57) Krainer, K.; Bressan, D.; Dietre, B.; Haas, J.N.; Hajdas, I.; Lang, K.; Mair, V.; Nickus, U; Reidl, D.; Thies, H.; Tonidandel, D. (2015b): A 10,300-year-old permafrost core from the active rock glacier: Lazaun, southern Ötztal Alps (South Tyrol, northern Italy). Quat. Res. 83, 324–335.

(58) Krainer, K.; Ribis, M.; Schmidt, V. (2015c): Der Blockgletscher im Inneren Hochebenkar. In: Schallhart, N.; Erschbamer, B. (Ed.): Forschung am Blockgletscher. Methoden und Ergebnisse. Innsbruck Univ. Press, Innsbruck (Alpine Forschungsstelle Obergurgl, 4), 77–96.

(59) Thies, H.; Nickus, U.; Tessadri, R.; Tropper, P.; Krainer, K. (2017): Peculiar arsenic, copper, nickel, uranium, and yttrium-rich stone coatings in a high mountain stream in the Austrian Alps. Austrian Journal of Earth Sciences 110 (2), 1–7. https://doi.org/10.17738/ajes.12017.10012.
